# Autophagy‐Targeting Nanomedicine: Strike at the Heart of the Cancer via Precise Modulation of Autophagy

**DOI:** 10.1002/EXP.20240112

**Published:** 2025-08-26

**Authors:** Zhouyi Sun, Huali Zuo, Kai Zhang, Yang Liu, Qianwen Wang, Qitao Hu, Jiwei Qian, Andreas Lundqvist, Bo Zhang, Weiyu Chen, Zhe Tang

**Affiliations:** ^1^ Department of Surgery The Fourth Affiliated Hospital, Zhejiang University School of Medicine Yiwu China; ^2^ Department of Respiratory and Critical Care Medicine, Center for Oncology Medicine, the Fourth Affiliated Hospital of School of Medicine, and International School of Medicine International Institutes of Medicine, Zhejiang University Yiwu China; ^3^ Key Laboratory of Surface & Interface Science of Polymer Materials of Zhejiang Province, School of Chemistry and Chemical Engineering Zhejiang Sci‐Tech University Hangzhou China; ^4^ Department of Oncology‐Pathology Karolinska Institute Stockholm Sweden; ^5^ Department of Surgery, The Second Affiliated Hospital Zhejiang University School of Medicine Hangzhou China; ^6^ Zhejiang Key Laboratory of Precision Diagnosis and Treatment for Lung Cancer Yiwu China; ^7^ Zhejiang university‐Sweden Joint Laboratory of Tumor Immunology Yiwu China

**Keywords:** Autophagy, Nanoplatform, Autophagy‐targeting nanomedicine, Cancer therapy, Combined therapy

## Abstract

Autophagy is a process of engulfing cytoplasmic proteins or organelles, thereby fulfilling cells’ metabolic needs and the renewal of specific organelles. Given its key roles in tumor progression, autophagy has attracted tremendous attention in cancer therapies. Notably, there is a megatrend to integrating autophagy regulation into mainstream treatments. This review focuses on autophagy‐targeting nanomedicine (ApT‐NM) to modulate autophagy in tumor therapy, including the unmodified and functionalized nanoparticles that target tumors by carrying autophagy modulators. On the one hand, it can reverse treatment resistance by inhibiting protective autophagy, and on the other hand, it can promote the death of cancer cells through type II apoptosis by inducing autophagy. Moreover, advanced nanoplatforms combining various treatments (such as chemotherapy, radiotherapy, photothermal therapy, and photodynamic therapy, etc.) have also been summarized. Last, the future perspectives and directions for ApT‐NM research are provided, hoping to emphasize this rising filed and promote the development of ApT‐NM.

## Graphical abstract

This review emphasizes the critical role of autophagy in cancer treatment, highlighting the development of autophagy‐targeting nanomedicines and their applications in combination therapies. It explores how nanoplatforms can effectively regulate autophagy, underscoring the potential of integrating autophagy modulation with other treatment strategies to enhance cancer treatment efficacy. The review offer valuable insights for developing more precise and effective anti‐cancer approaches.

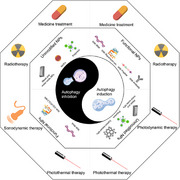

## 1 Introduction

Cancer has emerged as one of the top three causes of death globally [1]. Although various conventional cancer therapy methods have been well‐established, including surgical resection, chemotherapy, and radiotherapy, the therapeutic efficacy is still unsatisfactory [2]. Alternatively, targeting therapies via small molecules and immunotherapy, such as checkpoint inhibitors, are gradually applied in the clinic. However, drug resistance significantly limits the therapeutic effects of these treatments. Gene mutations in cancer cells can alter therapeutic targets, rendering drugs that were initially effective ineffective. For instance, mutations in the EGFR gene can prevent certain anti‐EGFR drugs from binding effectively [3]. Additionally, the tumor microenvironment contains immunosuppressive factors, such as tumor‐associated macrophages and regulatory T cells, which can suppress the immune response and increase the tumor's tolerance to immunotherapy [4]. Furthermore, some tumor cells can utilize autophagy to eliminate drug‐induced damage, thereby enhancing their survival potential. In addition, scientists keep developing novel treatments such as photothermal therapy (PTT), photodynamic therapy (PDT), and sonodynamic therapy (SDT) as optional medical technology [5]. Currently, there is limited research investigating these novel therapies, so they are not widely used clinically.

**FIGURE 1:**
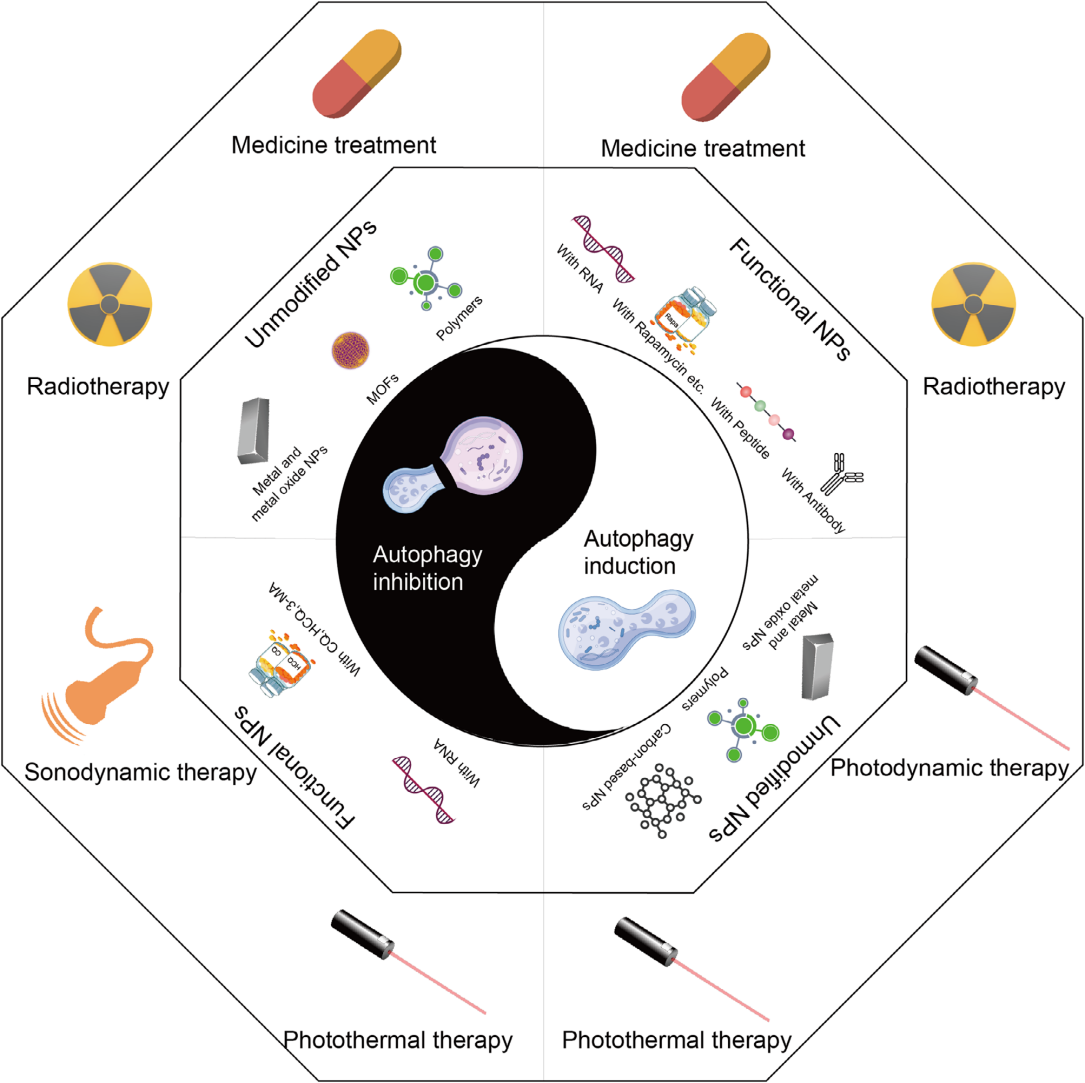
The combined therapeutic strategies of nanomedicine associated with autophagy modulation.

To enhance the therapeutic effect, autophagy drawn the attention of researchers. Autophagy plays a crucial role in maintaining cellular metabolism and homeostasis at a basal level. In the context of cancer, autophagy manifests as a dynamic process that can act both as an early‐stage tumor suppressor and as a late‐stage cancer promoter, contributing to tumor maintenance and treatment resistance. Cancer cells often show elevated levels of autophagy in response to various treatments. This autophagy induced by anti‐cancer therapy can be a pro‐survival or pro‐death mechanism, a crucial process determining the fate of cancer cells. Hence, many studies aim to anti‐tumor via regulating autophagy in cancer cells. This balance of autophagy modulation in tumor therapy can be likened to the traditional concept of “yin and yang,” highlighting the importance of understanding and maintaining equilibrium in this process (Figure 1). However, there are currently few strategies that can change autophagy directly, and regulators are challenging to exert the effects on cancer precisely without affecting normal cells and body organs.

Nanoscience has a profound history dating back to when Richard Feynman first demonstrated the concept of nanotechnology in 1959 [6]. Recently, a series of studies focused on biomedical nanotechnologies due to their considerable potential. Notably, cancer treatment has become one of the most intriguing fields of the application of nanoscience through combining different conventional and emerging therapeutic strategies [7]. Through passive or active targeting mechanisms, nanoparticles could control the therapeutic release in tumor sites accurately and reduce unexpected effects like harmful off‐target effects [8]. Thus, nanocarriers have been developed to improve anti‐cancer therapeutic effects while limiting the systemic toxic effects of antineoplastic agents. For example, since these NPs can function as carriers, they are involved in autophagy regulation via delivering modulators of autophagy, such as the delivery of beclin‐1 for breast cancer [9]. Otherwise, nanoparticles themself can act as a type of physical stimulation or chemical reagent that can influence autophagy. Then, the emergence of nanocarriers compensates for a series of therapeutic limitations by regulating autophagy.

In this review, we briefly describe the molecular mechanisms of autophagy and the relationship between autophagy and cancer first treatment therapy. More critically, we focus on the recent advances in nanomedicine targeting autophagy in cancer therapy, and discuss various strategies based on promoting autophagy or inhibiting autophagy, aiming to highlight these strategies for enhancing anti‐tumor efficacy as combined therapies. The combined therapeutic strategies of nanomedicine, associated with autophagy modulation (Figure 1).

## 2 Autophagy‐Targeting Nanoparticles Involved in Tumor Treatment

### 2.1 Progress of Autophagy in Cancer Cells

In the mid‐1950s, researchers made a noteworthy observation regarding a novel, highly specialized “compartment” within the cell (scientifically termed as an organelle). This organelle contained enzymes responsible for breaking down proteins, carbohydrates, and lipids. This distinct compartment, referred to as a lysosome, functions as a processing hub for decomposing cellular constituents. For his groundbreaking discoveries related to lysosomes and peroxisomes, the Belgian scientist Christian de Duve was awarded the Nobel Prize in Physiology or Medicine in 1974. Additionally, he bestowed the term “autophagy” to describe this cellular process.

**FIGURE 2:**
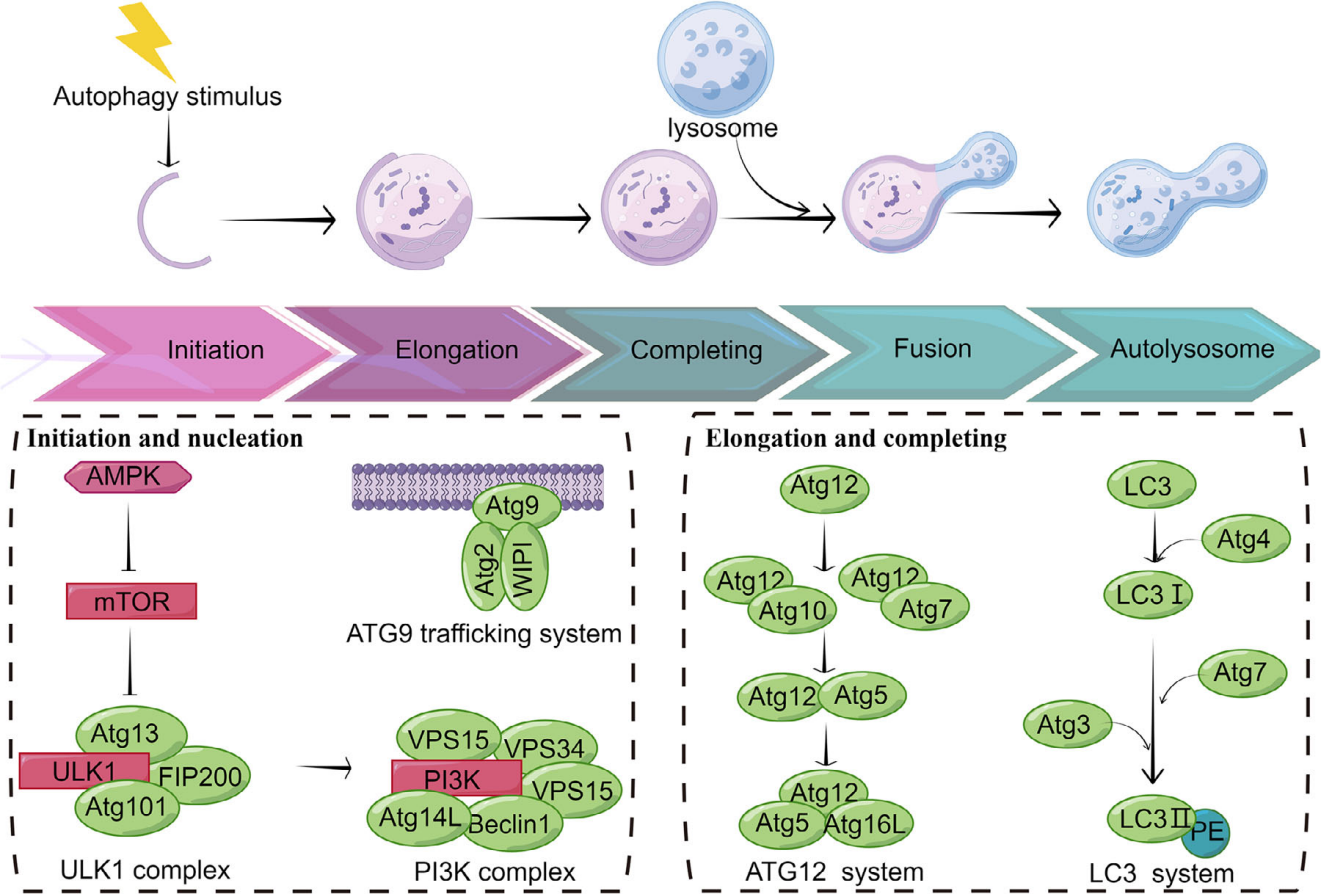
Schematic overview of autophagy in mammalian cells.

Autophagy, a cytoprotective catabolic process, can be effectively induced when the cellular environment changes under diverse stimuli. In these cases, autophagy can prevent cell injury and promote survival under conditions of energy or nutrient scarcity [10]. During cancer invasion, autophagy can be triggered as well. Under the attack of tumor factors, the excessive or damaged cytoplasmic materials can be transported to lysosomes for advanced degradation through one of the following mechanisms: micro‐autophagy, chaperone‐mediated autophagy, and macro‐autophagy [11]. Macro‐autophagy contains a series of complicated processes and can be modulated by related proteins like autophagy‐related genes (ATGs). ATGs play different roles in the membrane remodeling steps for the formation and maturation of autophagosome, a double‐membrane vesicle structure [12]. After swallowing the selected components, autophagosomes fuse with lysosomes‐autolysosomes, conjugating LC3 on the inner autophagosome membrane and the autophagy cargo receptors (ACRs). The phagocytosed cargo is subsequently degraded with the help of pH‐sensitive hydrolases and other programs such as ferroptosis, a form of autophagy‐dependent cell death [13]. After eliminating the components, the degraded cargo would be released, and lysosomes would be reformed to maintain the autophagic flux. Micro‐autophagy and chaperone‐mediated autophagy, considered selective autophagy, directly deliver the targeted cargo to the lysosome [14]. Here, we briefly present the mechanism of autophagy in mammalian cells. At least four types of biologically active products are involved in regulating autophagy. The process is detailed in Figure 2.

1. The unc‐51‐like kinase 1 (ULK1) complex. This complex includes ATG13, FIP200, ATG101 and plays an important role in autophagy initiation. It is biologically characterized by the regulation of adenylate‐activated protein kinase (AMPK) and mammalian cell target of rapamycin (mTOR) pathways [15].
2. The class III PI3K complex I. Among them. The components of P13K complex I include Vps34, Vps15, ATG6/Beclin1, ATG14L. This complex localizes to pre‐autophagosomal structure (PAS) or autophagy precursors in mammalian cells and can bind to membranes to catalyze the conversion of lipid molecules phosphatidylinositol (PI) to phosphatidylinositol 3‐phosphate (PI3P), thereby recruiting proteins to bind to PI3P [16].
3. The ATG9‐ATG2‐WIPI complex. ATG9 vesicles can circulate in the bilayer membrane and cytoplasm by interacting with ATG2‐WIPI complex, which is essential in membrane structure transport during autophagosome formation [17]. It could shuttle between PAS and peripheral organelles to deliver lipids/factors during phagophore expansion.
4. The ubiquitin‐like systems. ATG12 are ubiquitin‐like proteins that bind to lipid molecules phosphatidyl ethanolamine (PE) and ATG5, respectively [18]. LC3 has been deeply studied and characterized as an autophagosome marker in mammalian cells, which forms an LC3 binding system [19]. In addition, studies have reported the protein structures of ATG7 and ATG3, which define the ubiquitination‐like pathway at the molecular structure level [20].

Autophagy could act as a survival mechanism during the initiation and early stages of cancer progression. For instance, autophagy can digest damaged mitochondria to maintain harmony with genomic stability and cellular homeostasis [21]. In advanced phases or during cancer therapy, autophagy can promote the capability of tumor cells to endure harsh environments such as hypoxia and metabolic stresses and facilitate tumor metastasis [22]. Recent studies have also shown that autophagy aids immune evasion in pancreatic cancer by degrading MHC‐I [23].

### 2.2 Application of Autophagy Modulator in Cancer Therapy

#### 2.2.1 Reversing Treatment Resistance in Cancer Cells by Inhibiting Autophagy

Currently, drug inhibitors and gene intervention are the most commonly used methods for inhibiting autophagy, while numerous autophagy modulators are available, including 3‐methyladenine (3‐MA), chloroquine (CQ) and hydroxychloroquine (HCQ), etc.

Commonly used autophagy inhibitors in cancer treatment at present. 3‐methyladenine (3‐MA) is one of the earliest autophagy inhibitors employed in autophagy research. Its primary mechanism of action involves disrupting autophagic vesicle formation by inhibiting class I phosphatidylinositol 3‐kinase C3 (PI3K‐C3), thus interfering with autophagy initiation in the early stages. A novel chemotherapeutic drug CYT997 was utilized to induce apoptosis and autophagy in osteosarcoma cells. However, CYT997‐induced autophagy could promote cancer cell survival, and reinforcement of autophagy inhibitor 3‐MA could lessen the effect and result in a better anti‐tumor effect in vivo [24]. Seglen and Gordon described the action of 3‐MA, noting that it can completely inhibit autophagy at high concentrations but induces autophagy at low concentrations [25]. At low concentrations over an extended period, 3‐MA may promote autophagy by inhibiting the class I enzyme. Therefore, wortmannin or LY294002, both specific inhibitors of PI3‐kinase, are currently considered viable alternatives to 3‐MA.

Furthermore, chloroquine (CQ) and hydroxychloroquine (HCQ) are widely utilized in cancer autophagy research. A study employing CQ to block ER stress‐related autophagy significantly enhanced apatinib‐induecd apoptosis in CRC cell lines in vitro and in vivo [26]. However, HCQ was chosen as the autophagy inhibitor in clinical trials due to its lower toxicity at peak concentrations than CQ [27]. In a phase II clinical trial conducted by Arora, et al., participants were administered vorinostat (VOR) 400 mg and HCQ 600 mg orally daily, compared to regorafenib 160 mg orally daily with a schedule of 3 weeks on and 1 week off, repeated every 4 weeks, in patients with metastatic colorectal cancer [28]. The study found that the combination of VOR and HCQ resulted in alterations in anti‐tumor immunity with desirable safety. However, it did not lead to an extension in progression‐free survival (PFS) or translate into clinical efficacy. Currently, another phase II clinical trial is underway to investigate the safety and efficacy of the CDK4/6 inhibitor Palbociclib in combination with the HCQ for HR+HER2 breast cancer (NCT05953350).

Additionally, there are several discovered autophagy inhibitors. Deciphera Pharmaceuticals LLC is currently conducting a Phase I/II clinical study on a novel autophagy inhibitor, DCC‐3116 (NCT04892017). This represents a groundbreaking development as it is the first‐in‐class selective ULK1/2 autophagy inhibitor. Prior to this, its efficacy has been validated in various preclinical models and various cancers such as NSCLC, GIST, and so on when combined [29]. This trial will assess the effects of combining DCC‐3116 with trametinib, binimetinib, or sotorasib on patients with advanced or metastatic solid tumors harboring RAS/MAPK pathway mutations.

Using gene therapy to overcome autophagy‐induced therapeutic resistance is the current focus of intense research. The overexpression of autophagy‐suppressing genes by using circRNAs/lncRNAs or gene silencing to knock down Atgs by using small interfering RNAs (siRNAs) are two typical methods as gene interventions [30]. For instance, Zhang, et al. decreased the stability of serine and arginine‐rich splicing factor 6 (SRSF6) via overexpressing the target gene CRNDE. The process can mediate the inhibition of autophagy in gastric cancer cells to promote chemosensitizations of oxaliplatin and 5‐FU [30a]. Not only that, but a similar study also reported CircCUL2 inhibits autophagy by targeting miR‐142‐3p in cisplatin‐resistant gastric cancer (GC) cells. This circRNA was found to inhibit the intracellular autophagosome marker LC3, Beclin1, and to form fewer autophagic vesicles, inhibiting resistant GC cell growth and metastasis [30b].

As of the current state of affairs, there remains a considerable distance to traverse in translating the modulation of ATGs for autophagy regulation into clinical practice. This is due to the inherent differences among individuals, the intricate complexity of genetic networks within organisms, and the challenging feasibility of conducting clinical trials.

#### 2.2.2 Induction of Autophagy Enhances the Anti‐Tumor Therapeutic Efficacy

In comparison to inhibiting autophagy, inducing autophagy is notably more straightforward. Various forms of stress can potentially trigger autophagy, such as pharmaceutical treatments or physical interventions. Currently, the most commonly employed potent autophagy inducers in research are rapamycin and its derivatives. These compounds operate by impeding the mTORC1 pathway to initiate autophagy and find extensive application in the investigation of autophagy in both cellular and animal models. Clinical phase I trials using rapamycin to treat conditions such as neurological and hematological disorders have been initiated, demonstrating favorable tolerability and delivering consistent safety outcomes among patients [31]. However, clinical studies for cancer treatment with rapamycin have not yet commenced, and further preclinical research is required before proceeding with such investigations.

Gene intervention is also a commonly used method to induce autophagy. For instance, MicroRNA‐145‐3p caused MTORC1 inactivation by targeting HDAC4, leading to autophagy induction. This “induced” autophagy could promote multiple myeloma cell death. On this basis, combined MIR‐145‐3p‐bortezomib treatment caused an autophagy enhancement to a greater extent than single‐agent treatment in both in‐vitro and in‐vivo experiments. It subsequently led to more potent cell death [32]. Repression of the AURKA‐CXCL5 axis by siRNA can promote autophagic cell death and sensitize non‐small‐cell lung cancer cells to radiotherapy [33].

### 2.3 Dual Strategies in Anti‐Cancer Autophagy‐Targeting Nanomedicine

As a key factor for tumorigenesis and development, autophagy is a promising target in cancer therapy. Due to the dual role of autophagy in tumor development, two different therapeutic strategies can be developed. The first approach involves the targeted induction of autophagic cell death. Another strategy is to sensitize cancer cells to other therapies by inhibiting the cytoprotective effect of autophagy.

#### 2.3.1 Anti‐Cancer Nanomedicine via Inhibition of Autophagy

##### 2.3.1.1 Metal Nanoparticles and Metal Oxide Nanoparticles

Several nanomaterials can act as regulators in tumor therapy by inhibiting autophagy [34]. Metal and metal oxide nanoparticles are the most prominent, as they regulate reactive oxygen species (ROS), a key molecule in autophagy signaling [35].

Platinum‐Nano (Pt‐Nano), as a prodrug, induce oxidative activation via platinum atoms on the surface. Thus, it can be used to treat cisplatin‐sensitive and resistant tumors. More importantly, Pt‐Nano only triggers only mild autophagy compared to cisplatin, dramatically reducing tumor cells' drug resistance [34a]. Previous studies have also shown that TiO_2_ nanoparticles could exhibit dose‐dependent autophagy regulation in human keratinocytes (HaCaT cells). The autophagy was initiated at low doses (0.16 µg/ml), while overloading of TiO_2_‐NP at high doses (25.0 µg/mL) induced autophagy dysfunction [34b]. Azimee, et al. utilized TiO_2_‐NP to suppress autophagy and overcome the drug resistance of 5‐fluorouracil (5‐FU) in human AGS gastric cells, thereby enhancing the cytotoxicity and apoptosis via chemotherapy [34e].

Recent studies have combined Fe*_x_* O*_y_* and CeO_2−_ *_z_* nanoparticles as a selective lysosomal alkalizing agent [36]. This alkalizing agent is activated specifically in cancer lysosomes characterized by high concentrations of H^+^ and H_2_O_2_. It efficiently captures •OH and converts it to OH^−^, effectively damaging the enzymatic activity of cancer lysosomes and disrupting autophagy. The therapeutic efficacy of this approach has been validated in both primary and metastatic tumors models.

##### 2.3.1.2 Inorganic Nanoparticles

**FIGURE 3:**
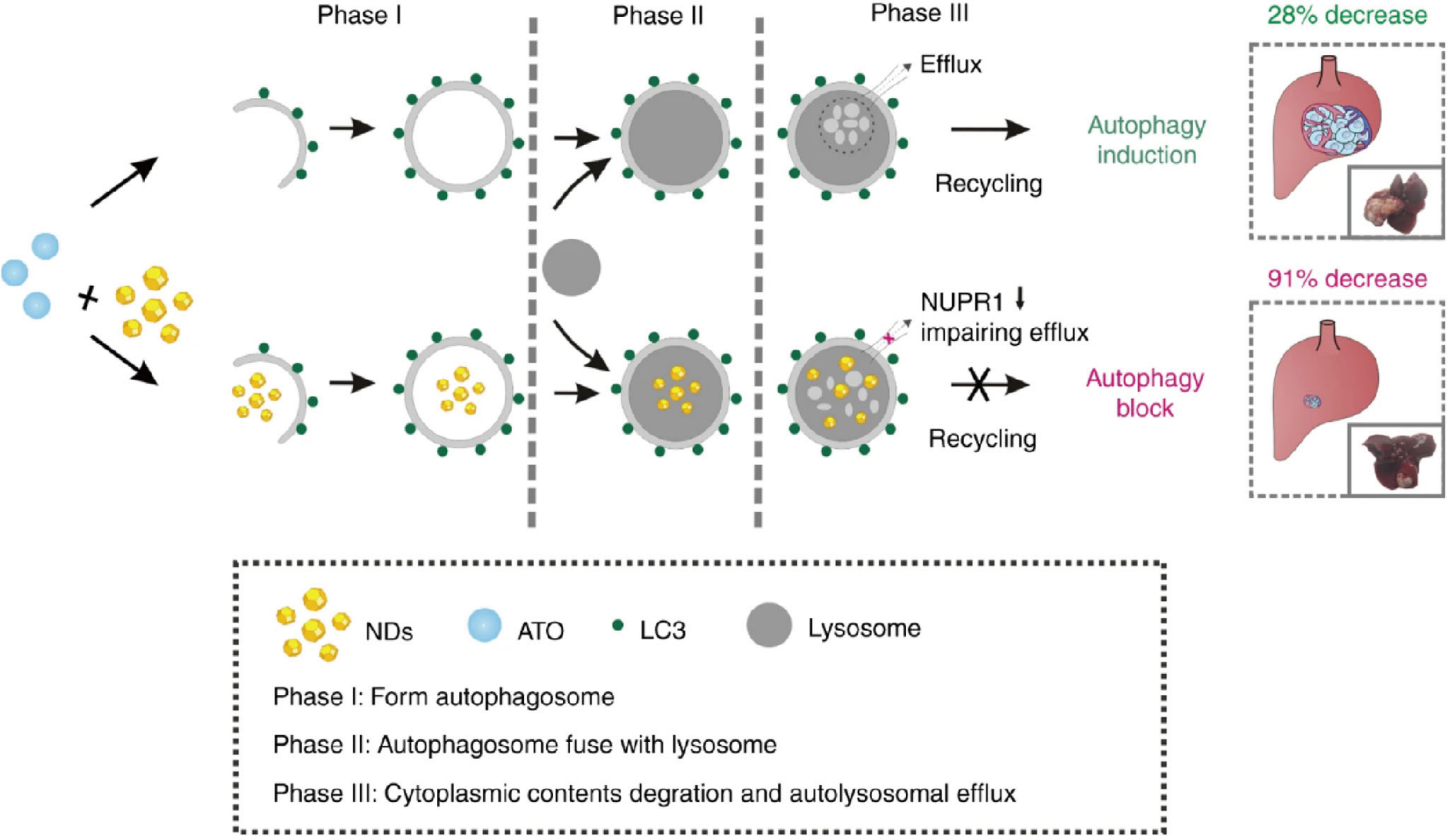
Molecular mechanisms underlying the enhanced therapeutic effect of NDs in ATO therapy for liver tumors and NDs improve the efficacy of ATO from 28% to 91% in tumor reduction through autophagy inhibition. Reproduced under the terms of the CC BY 4.0 license [34c]. Copyright 2018, Copyright Cui, et al.

Meanwhile, nano‐diamond (NDs) present an alternative approach to inhibiting autophagy in cancer cells [34c]. Compared with traditional chemical inhibitors (e.g., chloroquine or hydroxychloroquine) and metal nanoparticles (e.g., Fe_2_O_3_, Fe_3_O_4_, and AuNPs), NDs exhibit better autophagy inhibiting efficiency with desirable biocompatibility. Notably, NDs can selectively inhibit autophagy, with unique tissue specificity for tumors, liver and spleen. On this basis, Cui, et al. combined NDs with the chemotherapeutic drug arsenic trioxide (ATO) for liver cancer treatment. NDs reduce autophagy‐triggered drug resistance and improve chemotherapy for liver cancer, without any specific interaction with ATO, ensuring a safe dosage. In this way, side effects caused by arsenic accumulation were avoided, and ATO's efficacy was enhanced from 28% to 91% in tumor reduction (Figure 3).

Recently, another inorganic nanomaterial was shown to selectively inhibiting autophagy in tumor cells through an acid‐neutralization mechanism [37]. Layered double hydroxides, weakly alkaline nanoparticles, can be internalized by tumor cells, providing prolonged acid neutralization. This neutralization disrupts lysosomal or autolysosomal function, thereby inhibiting autophagy. Zhang, et al. also observed that Layered double hydroxides do not interfere with the lysosomal function of immune cells such as DC2.4 and RAW 264.7 while disrupting autophagy in cancerous cells, which may be related to the acidic tumor microenvironment.

##### 2.3.1.3 Organic Nanoparticles

The nanoscale size of these particles prolongs circulation and enhances tumor cell accumulation. However, naked nanomaterials generally act as an auxiliary role, and additional treatments are required to effectively prohibit autophagy in treating tumors [38].

**FIGURE 4:**
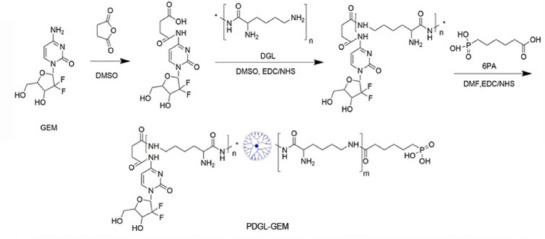
Schematic representation of the synthetic process for PDGL‐GEM. Reproduced under the terms of the CC BY 4.0 license [43]. Copyright 2021, Copyright Chen, et al.

A series of novel nanomedicines has been developed to exert stronger autophagy inhibition via further functionalizations, such as loading chemical autophagy inhibitors (e.g., chloroquine, 3‐methyladenine (3‐MA) and Lys05). Due to its high porosity and large surface area, metal‐organic frameworks (MOF) are commonly used as drug delivery carriers [39]. The crystal of zeolitic imidazolate framework (ZIF) are particularly notable in the MOF family. As a new type of nanoparticle, it has a high loading capacity and unique pH‐sensitive degradation properties [40]. For example, Shi, et al. designed CQ‐encapsulated ZIF‐8 nanoparticles to deliver CQ effectively in vivo [41]. In addition to CQ delivery, the excellent drug delivery and controlled release capabilities of the ZIF‐8 framework are also demonstrated in the encapsulation of 3‐MA [42]. ZIF‐8 can only disintegrate under low pH conditions, and the cumulative release of 3‐MA rapidly increased from pH 7.4 to pH 6.5 and pH 5.0 for 24 h, improving the autophagy inhibition efficiency of 3‐MA. Moreover, to exploit the acidic characteristics of the tumor microenvironment (TME), Chen, et al. developed a pH‐responsive “nano‐bomb” for pancreatic ductal adenocarcinoma (PDAC) treatment [43]. They used dendrigraft poly‐l‐lysine (DGL) modified with 6‐phosphonohexanoic acid (6PA) to generate PDGL, which was then loaded with gemcitabine (GEM), CQ and co‐precipitated with calcium phosphate (CAP) to form PDGL‐GEM@CAP/CQ (Figure 4). In the acidic tumor microenvironment, CAP “explodes” and dispersed CQ and PDGL‐GEM in TME. Then, CQ was internalized by surrounding fibroblasts, inhibiting the autophagy of fibroblasts from fibrosis and prohibiting metastasis. At the same time, PDGL‐GEM directly targets and kills tumor cells. The two parts of the “bomb” work synergistically to inhibit tumor growth and metastasis by inhibiting autophagy, thereby inhibiting tumor fibrosis and downregulating matrix metalloproteinase‐2 (MMP‐2), which further inhibits metastasis.

**FIGURE 5:**
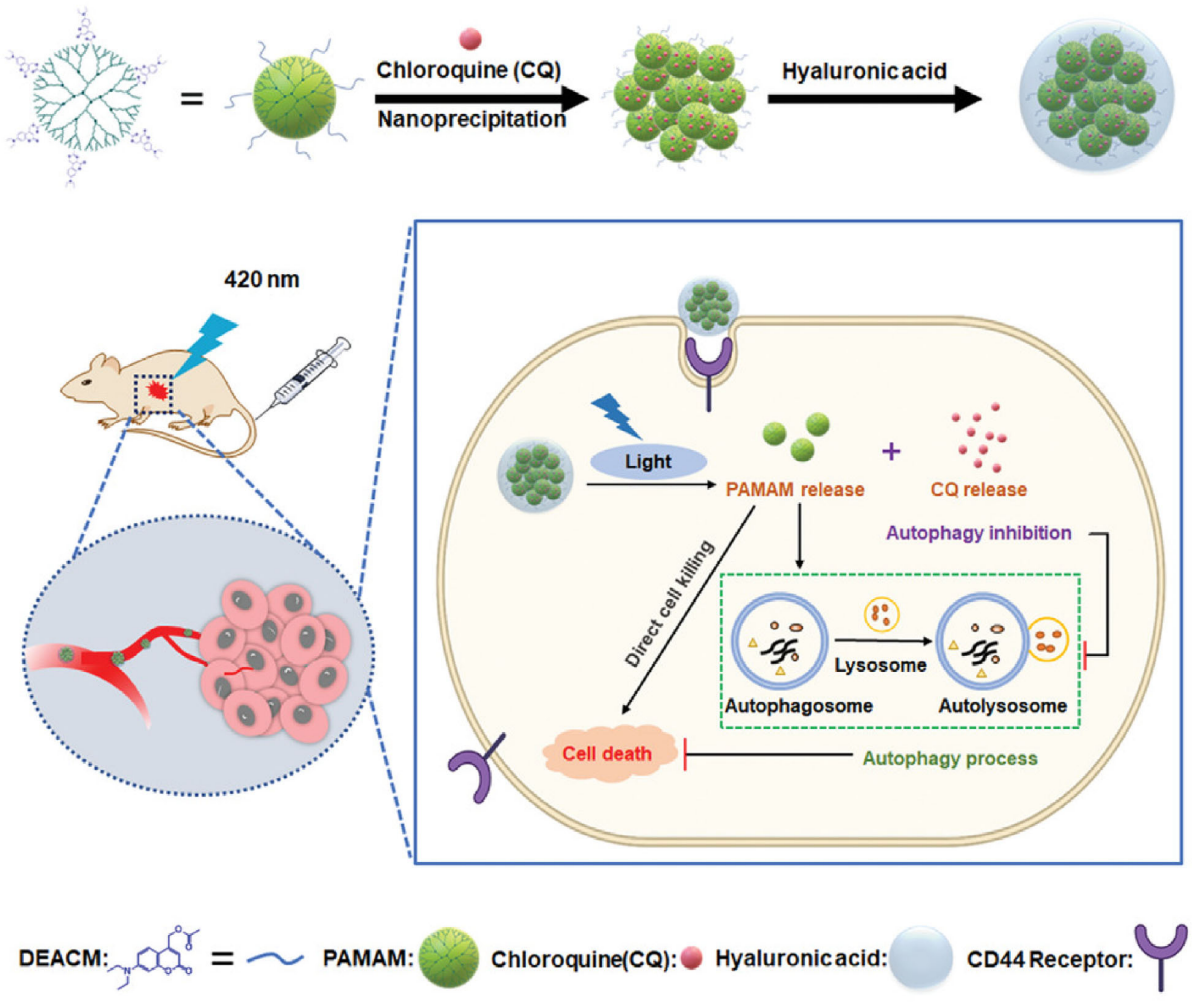
Schematic illustration of the preparation and mechanism of photoresponsive PAMAM‐assembled nanocarriers for synergistic cancer treatment, incorporating the loading of autophagy inhibitors. Reproduced with permission [44]. Copyright 2021, John Wiley and Sons.

Using a similar idea of environment‐responsive drug release, Jing, et al. designed a visible‐light‐responsive nanocarrier for drug delivery [44]. They selected the tree‐like generation 5 poly (amidoamine) (PAMAM G5) as a carrier to carry the autophagy inhibitor chloroquine. Unlike the ZIF‐8 framework described above, PAMAM itself is cytotoxic, and this study utilized this feature to directly kill tumor cells and induce tumor cells to produce pro‐survival autophagy. After the synthesis, CQ can be loaded inside PAMAM and externally coated with hyaluronic acid (HA). The layers constitute CQ@PD/HA, which enables the nanoparticles to have a prolonged circulation time and the ability to target CD44 receptors on the tumor surface. Then, with 420 nm irradiation at tumor site, CQ@PD/HA demonstrated cytotoxicity and autophagy inhibitory effect locally, which inhibited PAMAM‐induced autophagy and enhanced the killing effect (Figure 5).

Furthermore, some researchers have integrated the inhibition of autophagy with pyroptosis, a form of programmed cell death in cancer [45]. Deng, et al. employed PLGA nanoparticles coated with cancerous and mitochondrial membranes to encapsulate calcium ions and CQ. With cell membranes' homing effect and fusion mechanism, these nanoparticles can precisely recognize mitochondria and generate a substantial amount of ROS locally to induce pyroptosis in tumor cells selectively. Subsequently, they release CQ to inhibit mitochondrial autophagy, providing a combined treatment approach that enhances cellular pyroptosis through the dual effects of mitochondrial damage and autophagy inhibition. With the advantage in tumor targeting and mitochondrial‐selectively autophagy inhibition, the effectiveness of CQ was maximized.

##### 2.3.1.4 Self‐Assembly Nanomedicine

**FIGURE 6:**
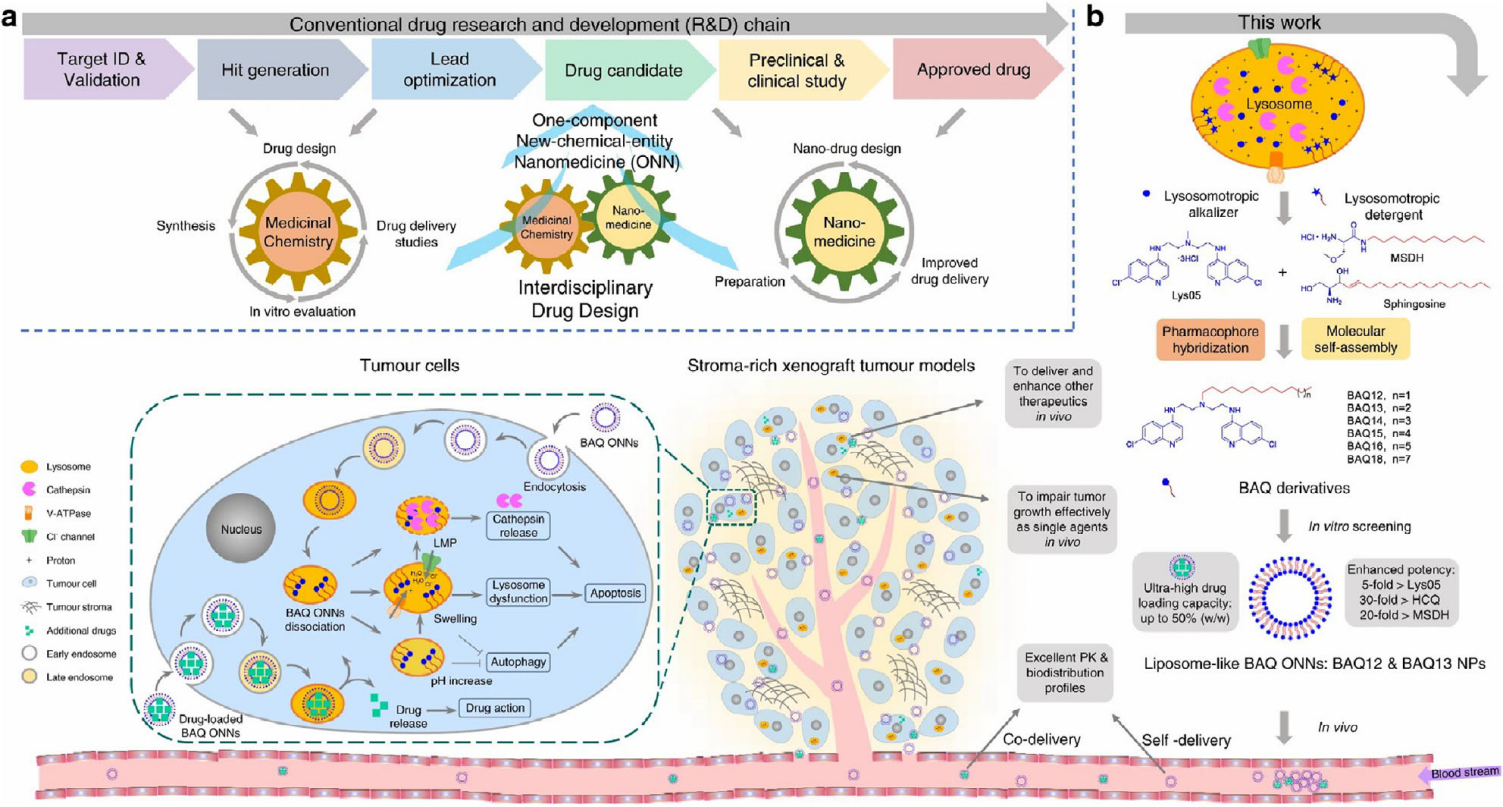
Schematic illustration of the proposed one‐component new‐chemical‐entity nanomedicines (ONNs) design strategy and the current work. Reproduced under the terms of the CC BY 4.0 license [46]. Copyright 2020, Copyright Ma, et al.

Unlike the traditional assembly approach of nanomedicine, Ma, et al. proposed a self‐assembly nano‐drug and developed a new one‐component new‐chemical‐entity nanomedicine (ONN) strategy [46]. This study combined Lys05, a BAQ bisaminoquinoline derivative, with lysosomotropic detergent (MSDH). As prepared, self‐assembled nanomaterials were highly influential in inducing lysosomal disruption, lysosomal dysfunction, and autophagy blockade, exhibiting 30‐fold stronger antiproliferative activity than CQ. In addition, other anti‐cancer drugs can continue to be encapsulated in the self‐assembled nanomaterials, making them play dual roles as therapeutic agents and delivery vehicles (Figure 6).

In conclusion, autophagy is one of the critical links in tumor occurrence and progression. Various types of nanomedicines inhibit autophagy through different mechanisms, such as ROS regulation, lysosomal disruption, or pH‐responsive drug release. Key examples and strategies are summarized in Table 1. However, due to the limited distribution, NPs can only inhibit autophagy within partial malignant tissues. As such, it is difficult to completely eliminate tumors by regulating autophagy alone. Prioritization of combination therapies is therefore indispensable to effective treatment.

**TABLE 1:** Representative nanomaterials that inhibit autophagy in anti‐tumor therapy.

Type of NPs	NPs	Size	Functional groups	Drug/gene delivery	In vitro/in vivo	Cell/animal model	Major outcomes	Ref.
Carbon‐based nanoparticles	NDs	2–10 nm	—	—	In vitro, in vivo	HepG2 and NB4 cells, orthotopic liver cancer transplantation model	NDs act as autophagy inhibitors by attenuating NUPR1‐mediated efflux of intracellular autolysosomes, thereby allosterically enhancing the tumoricidal effect of ATO in a liver cancer model	[34c]
Metal nanoparticles	Nano‐Pt	1 nm	—	—	In vitro	A2780 and A2780cis cells,	Nano‐platinum causes only mild autophagy, so it can greatly reduce or even eliminate the possibility of autophagy‐related drug resistance	[34a]
Metal oxide nanoparticles	TiO_2_ NPs	10–25 nm	—	—	In vitro	AGS cells	TiO_2_ NPs inhibit autophagy by increasing ROS levels, impairing the normal function of lysosomes, and directly interacting with key autophagy proteins	[34e]
	Lysosomal alkalizing nanoparticle (FexOy core and CeO_2_‐z satellite structure)	18.2 nm	—	—	In vitro, in vivo	LLC cells, HeLa cells, subcutaneous tumor model, orthotopic murine lung cancer model and metastatic murine lung cancer model	Lysosomal alkalizing nanoparticle acting as selective lysosomal alkalizers in tumor cells, inhibit lysosomal enzyme activity and autophagy by leveraging the high concentrations of H⁺ and H_2_O_2_, ultimately leading to cancer cell apoptosis.	[36]
Metalloid nanoparticles	SeNPs	58 nm	Laminarin polysaccharides	—	In vitro	HepG2 cells	LP‐SeNPs inhibit the late stage of autophagy by inhibiting lysosomal function in HepG2 cells and exacerbating the apoptosis of HepG2 cells	[34d]
Metal‐organic frameworks	ZIF‐8	250nm	FA‐PEG/CQ@ZIF‐8 NPs	CQ	In vitro	HeLa and HEK293 cells	NPs exhibit pH‐responsive CQ release behavior, which can transfer CQ into FR‐overexpressing HeLa cells for targeted delivery and controlled release of CQ	[41]
	ZIF‐8	90–100nm	3‐MA@ZIF‐8	3‐Methyladenine	In vitro, in vivo	HeLa cells, Xenograft nude mice	3‐MA@ZIF‐8 can lead to severe blockage of autophagosome formation and autophagic flux, showing significant antitumor effect in vitro and in vivo	[42]
Organic nanoparticles	lipophilic cationic BAQ derivatives	100–140 nm	—	lysosomotropic detergent (MSDH), autophagy inhibitor (Lys05)	In vitro, in vivo	MIA PaCa‐2 and HT29, subcutaneous xenograft models	BAQ ONN shows excellent anti‐cancer activity with enhanced effects on lysosomal disruption, lysosomal dysfunction and autophagy inhibition	[46]
Polymers	Polymeric nanoparticles	125.25 ± 1.06 nm	PDGL‐GEM@CAP/CQ	GEM, CQ	In vitro, in vivo	Pan 02 and NIH3T3 cells, Pan 02 xenografts model, orthotopic model	Low pH triggers NPs to release GEM and CQ deep into the tumor. CQ assists GEM in tumor killing and inhibits tumor fibrosis and downregulates MMP‐2 by inhibiting autophagy, which further inhibits metastasis	[43]
	Polymeric nanoparticles	92.4 nm	CQ@PD/HA	DEACM, CQ	In vitro, in vivo	MDA‐MB‐231, MCF‐10A, N2A, A549 and Hela cells, orthotopic MDA‐MB‐231 breast cancer model	CQ@PD/HA releases CQ in response to light and inhibits PAMAM‐induced pro‐survival autophagy	[44]

Abbreviation: NDs: nanodiamonds; ATO: arsenic trioxide; Nano‐Pt: nanoparticulate platinum; TiO_2_ NPs: titanium dioxide nanoparticles; SeNPs: selenium nanoparticles; ZIF‐8: zeolitic imidazolate framework; CQ: chloroquine; DEACM: 7‐diethylamino‐4‐hydroxymethylcoumarin; GEM: gemcitabine;.

#### 2.3.2 Anti‐Cancer Nanomedicine by Induction of Autophagy

Autophagy promotes cancer cells resistant to induced apoptosis, thereby protecting cancer cells against chemotherapy. However, sustained or excessive metabolic cycling of intracellular components can occur with high levels of autophagy, resulting in excessive removal of essential proteins and organelles, ultimately leading to caspase‐independent autophagic cell death that is beneficial for therapy [47]. Representative nanomedicines that induce autophagy and their underlying mechanisms are summarized in Table 2.

**TABLE 2:** Representative nanomaterials for inducing autophagy in antitumor therapy.

Type of NPs	NPs	Size	Functional groups	Drug/gene delivery	In vitro/in vivo	Cell/animal model	Major outcomes	Ref.
Carbon‐based nanoparticles	Graphene oxide	15 nm	Graphene oxide‐silver nanoparticle (GO‐AgNPs)	—	In vitro	SH‐SY5Y cells	GO‐AgNPs nanocomplexes can induce stronger autophagy and impair the normal function of cells	[55]
	Fullerene derivatives	2–100 nm	—	—	In vitro, in vivo	A549, H460, H1299 and ECs cells. zebrafish embryos	Ten different structural fullerene derivatives were synthesized and only structurally specific fullerenes were found to induce autophagy	[57]
Metal nanoparticles	AgNPs	9–19 nm	β‐cyclodextrin (β‐CD) coated AgNPs	—	In vitro	MCF‐7, MDA‐MB‐468 cells	Induction of autophagy enhanced the uptake of AgNPs, in addition, the effect of AgNPs on autophagy showed a time‐dependent manner. Enhanced autophagic flux was observed at early time points; prolonged exposure resulted in flux inhibition	[51a]
	AgNPs	10–30 nm	Aqueous leaf extract of A. obesum (AOAgNPs)	—	In vitro	MCF‐7 cells	AOAgNPs enhance intracellular ROS levels and induce DNA damage, apoptosis and autophagy	[51b]
	AgNPs	16–20 nm	—	—	In vitro	Ovarian cancer cells	Combining AgNPs with salinomycin has the potential to enhance treatment efficacy in ovarian cancer by inducing autophagy.	[52]
Metal oxide nanoparticles	TiO_2_ nanoparticles	22.07±8.93 nm	—	—	In vitro	BEAS‐2B cells	Downregulation of Bcl‐2 expression by overexpressing miR34a enhances TNP‐induced autophagy, which then leads to higher levels of cell death	[48]
	ZnO NPs	50 nm	—	—	In vitro	U−2OS、MG−63、Saos−2 and 143B cells	The uptake of ZnO NPs induces the accumulation of autophagosomes and impairs lysosomal function, promoting the early and late stages of autophagy, respectively. Autophagy further triggers the release of zinc ions from ingested ZnO NPs, generating ROS to inhibit cell proliferation through S‐phase arrest	[49]
	ZnO NPs	30 ± 3 nm	ZnO‐MTCP	—	In vitro	MCF‐7, MDA‐MB‐468 cells	ZnO‐MTCP NPs increasing of the cytosolic calcium resulting in lysosomal and autophagy dependent cell death	[50a]
	ZnO NPs	20 nm	—	—	In vitro	SKOV3 cells	ZnO NPs lead to increased LC3 levels, mitochondrial damage, and autophagic death by inducing increased ROS	[50b]
	ZnO NPs	50 nm	—	—	In vitro	CAL 27 cells	ZnO NPs activate PINK1/Parkin‐mediated mitophagy in CAL 27 cells and promote tumor cell death	[50c]
	ZnO NPs	28.42 nm	Clausena lansium (Lour.) Skeels ZnO NPs	—	In vitro	SH‐SY5Y cells	Clausena lansium (Lour.) Skeels‐stripped ZnONPs induce autophagy and apoptosis in SH‐SY5Y neuroblastoma cells	[50d]
Polymers	Polymer nanoparticles	44.17–148.79 nm	Poly (β‐amino ester) copolymers	—	In vitro	MCF‐7 cells	Changes in the concentration of NPs caused different autophagic effects. Low concentrations of NPs induced autophagy in an mTOR‐dependent manner, but high doses of NPs resulted in autophagic cell death	[62]
	Polymer nanoparticles	97±3 nm	Amphiphilic triblock copolymer polyethylene glycol‐ploythymine‐poly(lactic‐co‐glycolic acid)	Aptamer AS1411, anti‐PFKFB4 siRNA, rapamycin	In vitro, in vivo	4T1 and HEK 293 cells, 4T1 TNBC xenograft tumor model	Novel ARPNPs are delivered to tumors in response to GSH release, downregulation of PFKFB4 helps to inhibit the SRC3/Akt/mTOR pathway, and combined with rapamycin shows the potential to induce tumor autophagy, in situ tumor destruction and tumor immune activation	[124]
	Polymer nanoparticles	—	—	Gold‐quercetin	In vitro, in vivo	Caski, Hela and Siha cells, xenograft tumor models	Quercetin nanoparticles can induce apoptosis, autophagy and proliferation inhibition of cancer cells through STATs‐regulated Bcl‐2/Caspase‐3 signaling pathway and PI3K/AKT‐related GSK and mTOR pathways	[125]
	Polymer nanoparticles	—	—	Gold‐quercetin	In vitro, in vivo	U87 and normal astrocytes, xenograft tumor models	Quercetin nanoparticles induced autophagy contributing to cell death by PI3K、AKT and mTOR pathway.	[126]
	Polymer nanoparticles	41 nm	NP‐B‐OVA	Autophagy‐inducing peptide beclin1	In vitro, in vivo	Bone‐marrow‐derived dendritic and B16‐F10 cells, B16‐F10‐OVA tumor‐bearing female C57/BL6 mice	NP‐B‐OVA enhances DC‐associated immune responses by upregulating autophagy and leads to enhanced antigen cross‐presentation, stimulates T cells and enhances antitumor immunity	[127]

Abbreviation: ROS: reactive oxygen species; MTCP: meso‐tetra (4‐carboxyphenyl) porphyrin); GSH: glutathione.

##### 2.3.2.1 Metal‐Based NPs

As mentioned above, different doses of TiO_2_‐NPs (TNPs) exert various autophagy effects [34b]. Low‐dose metal‐based NPs have been shown to induce autophagy, we may view these NPs as autophagy activators with anti‐cancer effects. Bai, et al. investigated possible molecular mechanisms, and they found that autophagy activation could also be considered a stressful process of clearance of the TNPs for cells via downregulating the miR‐34a and increasing expression of Bcl‐2 [48].

ZnO has also been reported to induce both early and late autophagy and suppress osteosarcoma cell proliferation by causing S phase arrest and inducing cell death due to both apoptosis and autophagy [49]. He, et al. further proved that autophagy could enhance zinc ion release from the uptaken ZnO NPs, and trigger osteosarcoma cell death with a high concentration of intercellular zinc ions. This effect can be reversed by antagonizing zinc ions with CaCl_2_ and EDTA. In the earliest steps of autophagy, autophagy and apoptosis may cooperatively induce cell death. In contrast, the late stage of autophagy may reverse cell deaths caused by apoptosis. Other studies also examined the mechanisms of autophagic cell death induced by ZnO_2_, such as increasing the cytosolic calcium resulting in autophagy‐dependent cell death, generating ROS and oxidative stress, and so on [50].

A similar effect was observed in AgNPs [51]. Thus, Zhang, et al. found that combining AgNPs with salinomycin may improve treatment efficacy in ovarian cancer by inducing massive autophagy [52]. They employed a novel bacterium called Bacillus clausii for the synthesis of silver nanoparticles (AgNPs). The synthesized AgNPs exhibited a uniform spherical shape with an average size of 16‐20 nm. This study identified a synergistic effect between AgNPs and Sal in inducing autophagy. While AgNPs alone had minimal impact on the expression of ATG3, ATG5, ATG6, ATG7, ATG10, ATG12, and ATG17, except for ATG5 and ATG7, Sal clearly upregulated all the tested genes, including ATG3, ATG5, ATG7, ATG12, and ATG17, except for ATG6 and ATG10.

NiO‐NPs are also capable of inducing autophagy in human cancer cells. In the study conducted by Cho, et al., it was found that NiO‐NPs enhance autophagic flux by generating intracellular reactive oxygen species (ROS) through mitochondria and subsequently activating the JNK pathway [53]. This autophagy modulation mediated by NiO‐NPs was associated with the induction of cell apoptosis.

##### 2.3.2.2 Nanocarbon‐Based Materials

**FIGURE 7:**
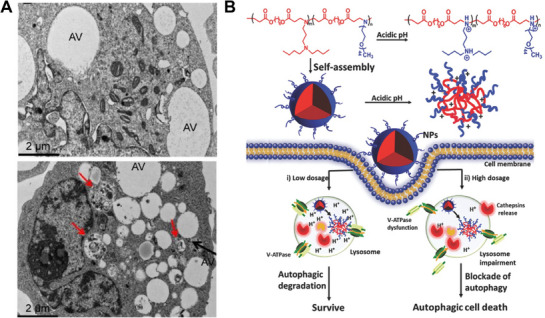
(A) The cells were treated with (up) GO (25 µg/mL), (down) GO‐AgNPs (5 µg/mL) for 24 h and processed for TEM. Reproduced under the terms of the CC BY 3.0 license [55]. Copyright 2017, Copyright Yuan et al. (B) Schematic illustration displayed autophagic effects of pH‐sensitivity polymeric NPs. Reproduced with permission [62]. Copyright 2016, John Wiley and Sons.

Carbon has various allotropes, so nanocarbon‐based materials usually contain numerous types, such as graphene, carbon dots, fullerene, etc. In previous studies, graphene oxide nanoparticles have been proven to induce autophagic vacuoles in neuroblastoma cell lines [54]. Yuan, et al. used graphene oxide (GO) and AgNPs to prepare nanocomposite [55]. As addressed above, AgNPs can be thought of as autophagy inducers. On electron microscopy, treatment with GO produced intensive cytoplasm vacuolization and significant numbers of mitochondria. The GO‐AgNPs‐treated cells showed many multi‐vesicular and membrane‐rich autophagosomes and a significant accumulation of autophagic vacuoles (Figure 7A). The nanocomposite induces greater initial oxidative stress and mitochondrial damage, further activating the autophagy process in neuroblastoma cells.

Fullerene (or a fullerene derivative), similar to graphene, was also shown to induce autophagy via the production of ROS and damage to mitochondria and the ER [56]. However, in the study by Wong, et al., ten different structural fullerene derivatives were synthesized, and only structurally specific fullerene derivatives containing amino acid residues (i.e., compounds with C─N bonds) were found to induce autophagy [57].

##### 2.3.2.3 Polymer Nanoparticles

There are several types of polymers, which can be composed of poly (lactic‐*co*‐glycolic acid) (PLGA), chitosan (CS), lipids, and so on [58]. Polymeric nanoparticles may play an important role in cancer therapy via exerting drug loading, and tumor‐targeting effect [59]. Besides, polymeric nanoparticles were always designed to respond to tumour area stimuli, increasing the anti‐tumor effect [60].

Lin, et al. designed a type of pH‐sensitive micelle‐like nanoparticles (NPs) that self‐assembled from poly (β‐amino ester)s [61]. These nanoparticles can enter into cancerous cells and accumulate in acidic lysosomes, inhibiting autophagosome‐lysosome fusion, leading to lysosome damage and enhancing ROS production to induce autophagy and apoptosis. Subsequently, they first systematically investigated autophagic effects of four types of polymeric NPs [62]. They found that different physical properties may cause other autophagy effects, especially the pH‐sensitivity, and low concentration of NPs‐induced autophagy, while a high dose of NPs blocked autophagic flux and resulted in autophagic cell death (Figure 7B).

## 3 Unveiling the Potential of Targeted Autophagy Nanoparticles in Cancer Combination Treatments

In the dynamic landscape of cancer therapeutics, the integration of nanomedicine with autophagy modulation has emerged as a promising avenue to transform treatment strategies. By harnessing nanotechnology to manipulate autophagy, a multifaceted approach is presented to enhance the effectiveness of combined therapies against cancer. This section review the complex interplay between autophagy and nanomedicine, exploring its potential to overcome resistance, sensitize tumors to radiotherapy, and optimize various therapeutic modalities such as photothermal, photodynamic, and sonodynamic therapies.

### 3.1 Autophagy‐Inhibiting Nanomedicine Recruited for Combined Therapies

**FIGURE 8:**
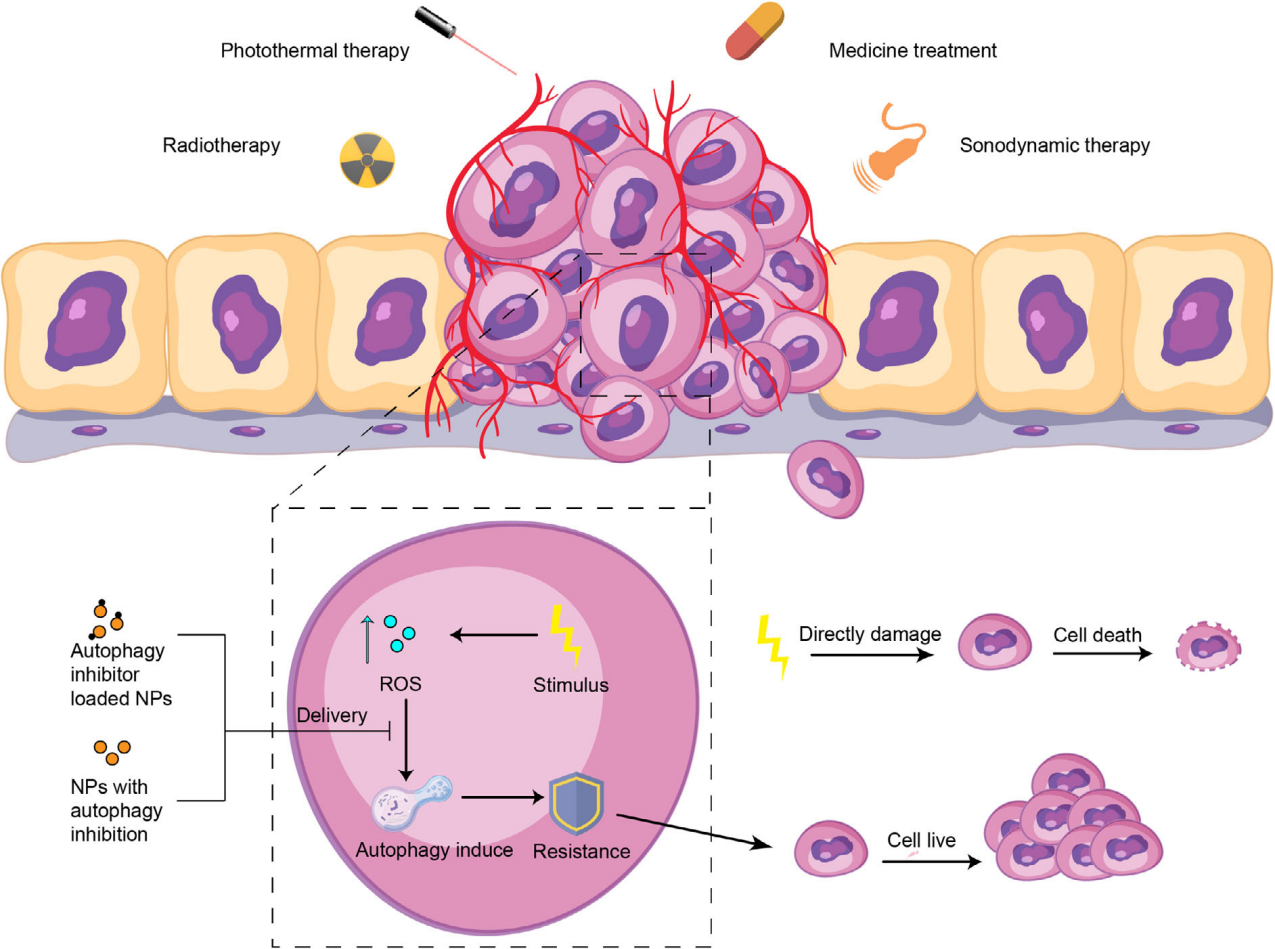
Schematic of combined treatment of anti‐tumor with nanoparticles inhibiting autophagy.

The utilization of autophagy‐inhibiting nanomedicine is a novel approach to enhance current treatment strategies. Cancer cells frequently use autophagy to resist destruction, hindering successful treatment. Current research mainly explores how inhibiting autophagy in nanomedicine can overcome drug resistance and make tumors more susceptible to traditional therapies like radiotherapy, as well as improve the effectiveness of PTT, PDT, and SDT (Figure 8). A detailed summary of multifunctional nanomedicines designed for autophagy inhibition in combination therapies is presented in Table 3.

**TABLE 3:** Representative autophagy‐inhibiting nanomedicine recruited in combined therapies.

Type of NPs	NPs	Size	Drug/gene delivery	In vitro/in vivo	Cell/animal model	Combination therapy	Major outcomes	Ref.
Carbon‐based nanoparticles	NDs	2–10 nm	—	In vitro, in vivo	HepG2 cells, orthotopic liver cancer transplantation model	Chemotherapy	NDs improve the ATO‐based therapy through increasing accumulation of LC3‐II and an autophagy substrate p62	[34c]
	NDs	100–130 nm	ICG, DOX	In vitro, in vivo	MDA‐MB‐231 cells, Xenograft nude mice	Chemotherapy, Photothermal therapy	NDs alleviate tumor thermal and drug resistance by inhibiting autophagy	[67]
Inorganic nanoparticles	Calcium phosphate nanoparticles	125 nm	CQ, doxorubicin	In vitro, in vivo	4T1 cells, orthotopic tumor murine model	Chemotherapy	NPs release CQ under acidic conditions and inhibit autophagy‐dependent degradation of post proteins, thereby inhibiting tumor metastasis and promoting the anti‐tumor effect of DOX	[71b]
	Lipid‐coated SiO2 NP	126 nm	HCQ, palbociclib	In vitro, in vivo	PANC‐1 and MIA PaCa‐2 cells, subcutaneous and orthotopic PANC‐1 models	Targeted therapy	The use of HCQ to inhibit palbociclib‐induced autophagy is demonstrated promising synergy	[72]
	HMSNs	50 nm	HCQ	In vitro, in vivo	HCT116 cells, HCT116 xenograft colon cancer models	Radiotherapy	HCQ‐HMSNs preferentially accumulate in tumor tissues and enhance the outcome of radiotherapy by blocking radiation‐induced autophagy.	[83]
	SiO_2_	190 nm	Bismuth, CQ	In vitro, in vivo	4T1 cells, Female BALB/c mice injected 4T1 cells	Photothermal therapy	NIR irradiation stimulates the release of CQ in nanomaterials, inhibits photothermal therapy‐induced autophagy, and enables anti‐tumor effects under milder conditions	[89b]
	Calcium phosphate nanocomposites	169.3 nm	DTX, CQ	In vitro, in vivo	MDA‐MB‐231 cells, Xenograft nude mice	Chemotherapy, Photothermal therapy	PCNPs/DC would enhance DTX‐mediated chemotherapy and PDA‐mediated PTT through complementary autophagy inhibition	[89d]
Lipid‐based nanoparticles	Nanoliposomes	143.2 nm	3‐Methyladenine	In vitro, in vivo	MCF‐7, MCF‐7 tumor‐bearing female BALB/c nude mice	Sonodynamic therapy	Nanoliposome delivery of autophagy inhibitors markedly reduced cellular resistance to intracellular oxidative stress and had a significant synergistic effect on apoptosis of cancer cells treated with SDT drugs.	[100]
Metal nanoparticles	AuNPs	38.10 ± 1.6 nm	HCQ, DOX	In vitro, in vivo	C6 cells, glioma‐bearing mice	Chemotherapy, immunotherapy	AuNPs release HCQ and DOX in the tumor area in response to legumain, and HCQ can inhibit DOX‐induced cytoprotective autophagy, thereby resensitizing glioma cells to DOX	[71c]
	Gold nanospikes	114 ± 40nm	—	In vitro, in vivo	KB cells, U14 xenograft tumor model	Radiotherapy	Appropriate combined use of autophagy inhibitors and gold nanostructures may be a useful strategy to increase the sensitivity of cancer cells to ionizing radiation in cancer radiotherapy.	[79]
	AgNPs	420 nm	—	In vitro	U251 cells	Radiotherapy	ROS plays an important role in autophagy induction and radiosensitization, and inhibiting the autophagy‐induced increase in ROS levels can further improve the effect of radiotherapy.	[80]
	Fe3O4@Ag nanoparticles	10.59 ± 1.07 nm	—	In vitro	U251 cells	Radiotherapy	Fe3O4@Ag nanoparticles can act as high‐performance radiosensitizers by reducing cytoprotective autophagy and increasing calcium‐dependent apoptosis.	[81]
	Core–shell copper selenide coated gold nanoparticles (Au@Cu_2−_ *_x_* Se NPs)	21.3±0.9 nm	—	In vitro, in vivo	U‐87MG cells, orthotopic glioblastoma athymic nude mice	Radiotherapy	Au@CS NPs could ameliorate the destructive effect of X‐ray irradiation on tumor cells by upregulating LC3‐II and SQSTM1/p62 protein levels and inhibiting their protective autophagy.	[82]
	Copper (Cu)‐palladium (Pd) alloy tetrapod nanoparticles	50.7–72.1 nm	—	In vitro, in vivo	4T1, MCF7, MDR and Hela cells, orthotopic xenograft models	photothermal therapy	TNP‐1 with the ability to induce pro‐survival autophagy can achieve superior therapeutic efficacy against drug‐resistant cancers through the combination of PTT and autophagy inhibitors	[89e]
	Metal‐DNA nanocomplexes (DACs‐Mn)	295 nm	DNA	In vitro, in vivo	4T1 cells, 4T1 subcutaneous tumors	Immunotherapy	DACs‐Mn consists of the AS1411 aptamer and manganese (Mn^2^⁺), enabling targeted delivery to tumor cells and the generation of reactive oxygen species. The inclusion of the ATG5 DNAzyme sequence inhibits autophagy, further promoting immunogenic cell death and enhancing the efficacy of immunotherapy.	[69]
Metal oxide nanoparticles	TiO_2_ NPs	10‐25 nm	—	In vitro	Human gastric adenocarcinoma AGS cell line	Chemotherapy	TiO_2_ NPs promote the cytotoxic and apoptotic effects of chemotherapy by increasing ROS levels and impairing the normal function of lysosomes	[34e]
	ZnO NP	—	—	In vitro, in vivo	SGC7901, BGC823, and SGC7901/DDP cell lines, Xenograft nude mice	Chemotherapy	ZnO NP alleviates the chemoresistance of GC cells by inhibiting autophagy	[68b]
	Superparamagnetic iron oxide nanoparticles	50 nm	MiR‐376B	In vitro, in vivo	HEK293T, MCF7, SKBR3 and MDA‐MB‐453 cells, xenograft nude mouse model	Chemotherapy	Targeted delivery of MIR376B successfully downregulated autophagy‐related targets of miRNAs and significantly increased the anti‐cancer activity of cisplatin	[74c]
	MnO_2_ nanoparticles	55.35 nm	CQ	In vitro, in vivo	T24 cells, T24 tumor bearing mice	Radiotherapy	HMCQ NPs can modulate the abnormal tumor microenvironment, restore the autophagy‐inhibitory activity of chloroquine, and greatly enhance the efficacy of radiotherapy	[84]
	IONP	32.1 ± 1.3 nm	—	In vitro, in vivo	MCF‐7 and LC‐3 cells, Nude mice‐bearing MCF‐7 xenograft	Photothermal therapy	IONP can induce significant autophagy in MCF‐7 cells under laser irradiation, and the combined inhibition of autophagy with chloroquine diphosphate can enhance its antitumor photothermal cytotoxicity in vitro and in vivo	[89a]
	Hollow mesoporous titanium dioxide nanoparticles	131.7 ± 1.6 nm	HCQ	In vitro, in vivo	MCF‐7, MCF‐7 tumor bearing BALB/c nude mice	Sonodynamic therapy	HCQ released in response to ultrasound stimulation is able to block autophagic flux and cut off nutrient supply from damaged organelles, which is expected to eliminate cellular resistance to SDT	[99]
Polymers	Polymeric nanoparticles (PEG‐PLA)	110 nm	CQ, DOX or DTX	In vitro, in vivo	MDA‐MB‐231 and MCF‐7 cells, MDA‐MB‐231 orthotopic tumor murine model	Chemotherapy	Co‐delivery of autophagy inhibitor chloroquine (CQ) and chemotherapeutic drugs (DOX or DTX) can significantly increase drug accumulation in tumor tissues and CSCs, and showed significant tumor inhibition while decreasing the proportion of breast CSCs	[71a]
	Poly(*N*‐isopropylacrylamide)	—	3‐MA, DOX	In vitro, in vivo	B16F10 and MCF‐7 cells, melanoma‐bearing C57BL/6 mice	Chemotherapy	Carrying the autophagy inhibitor 3‐MA improves the internalization of nanoparticles and enhances their therapeutic effect	[71d]
	Polymeric nanoparticles	54.44 ± 11.13 nm	Si‐beclin1, tetravalent cisplatin (Pt(IV))	In vitro, in vivo	A549 cells, A549 tumor‐bearing athymic nude mice	Chemotherapy	siBec1@PPN delivers cisplatin prodrug and inhibits autophagy via Beclin1 RNAi mechanism, further enhancing cisplatin chemotherapy and reversing drug resistance	[74b]
	Chitosan nanoparticle	106.5 ± 2.3 nm	shATG‐5, gefitinib	In vitro, in vivo	A549 and PLC cells, subcutaneous xenograft nude models	Chemotherapy	Co‐delivery of shATG‐5 and gefitinib loaded in CS NPs triggered the apoptosis pathway and enhanced synergistic antitumor effects via autophagy blockade.	[74d]
	Polymeric nanoparticles	75.7 ± 7.3 nm	ICG, PQ	In vitro, in vivo	MCF‐7 cells, MCF‐7 tumor xenograft models	Photothermal therapy	Inhibition of autophagy by PQ significantly improves the efficacy of the ICG‐induced photothermal tumor therapy by blocking autophagolysosome formation and enhancing apoptosis under laser irradiation	[89c]
	PDA nanoparticles	105 ± 45 nm	CQ	In vitro, in vivo	NIH3T3 and GFP‐LC3/HeLa cells, BALB/c nude mice bearing MDA‐MB‐231 tumors	Photothermal therapy	Inhibition of autophagy in tumor cells significantly enhanced the efficacy of PTT, resulting in complete tumor suppression at mild treatment temperatures	[92c]
	Nanocore of PDA and the nanoshell of hollow mesoporous silica	234.77 nm	CQ, glucose consumer glucose oxidase (GOx)	In vitro, in vivo	HepG‐2, HepG‐2 tumor‐bearing mice	Photothermal therapy	GOx‐mediated tumor starvation directly represses HSP expression, resulting in enhanced PDA nanocore‐induced low‐temperature PTT, and the inhibition of autophagy by the released CQ compensates for the efficiency loss of low‐temperature PTT, achieving an enhanced therapeutic effect	[93]
Semiconductor nanoparticles	Ag2S quantum dots	14.8±2.0 nm	5‐FU, cetuximab	In vitro	EGFR‐overexpressing A549 and EGFR low‐expressing H1299 cells	Chemotherapy, targeted therapy	mPEG–Ag2S–Cet/5‐FU may eliminate the necessity of adjuvant therapy since it showed suppression of 5‐FU‐induced autophagy by itself	[68a]
Supramolecular nanoparticles.	Based plectin‐1‐targeting peptide chimeric	180 ± 17 nm	MiR‐9	In vitro, in vivo	CFPAC‐1, PANC‐1, CAPAN‐1, NCI‐H1299 and A549 cells, Xenograft nude mice	Chemotherapy	miR‐9 enhances the sensitivity of pancreatic ductal adenocarcinoma to doxorubicin chemotherapy through the autophagy pathway	[74a]

Abbreviation: NDs: nanodiamonds; ATO: arsenic trioxide; CQ: chloroquine; ICG: indocyanine green; DOX: doxorubicin; HCQ: hydroxychloroquine; DTX: docetaxel; PTT: photothermal therapy; SDT: sonodynamic therapy; ROS: reactive oxygen species; IONP: iron oxide nanoparticle; HMSNs: hollow mesoporous silica nanoparticles; 3‐MA: 3‐methyladenine; PQ: primaquine; PDA: polydopamine; 5‐FU: 5‐fluorouracil.

#### 3.1.1 Overcoming Autophagy‐Induced Resistance Against Treatments

Clinically, chemotherapy, targeted therapies, immunotherapy remain the mainstream approaches for cancer treatment. Autophagy that leads to treatment resistance is induced by above three therapies [63]. Under such circumstances, combining autophagy inhibitors will reverse multidrug resistance [64]. Considering these advantages, utilizing nanoparticles as a combination drug delivery platform could exhibit tumor‐specific therapeutic effects. More than that, NPs may reduce the doses needed for agents, with consequent reductions in undesirable adverse effects and toxicity [65].

A growing body of work shows that various types of NPs can regulate autophagic responses in vivo and in vitro [66]. We refer to nanoparticles that inhibit autophagy as nanoparticle autophagy inhibitors (NAPI). As described previously, Cui, et al. compared the effects of a series of NPs on autophagy in HepG2 cells [34c]. Subsequently, they demonstrated that NDs function as autophagy inhibitors via impairing NUPR1‐mediated autolysosomal efflux in cells and used the mechanism in combination with chemotherapy agent arsenic trioxide (ATO). Combination therapy allosterically enhances the tumoricidal effect of ATO. A similar study utilizing NDs‐medicated autophagy inhibition augmented mild‐temperature photothermal and chemo combination therapy in triple‐negative breast cancer [67]. In addition to NDs, other nanoparticles have been reported to enhance chemotherapy efficacy through inhibiting autophagy, such as Ag_2_S quantum dots (QDs), zinc oxide nanoparticles (ZnO‐NP) and TiO_2_ NPs [34, 68]. Interestingly, most of these nanoparticle autophagy inhibitors are metal elements. Gu, et al. developed metal‐DNA autophagy‐targeted nanocomplexes to combine autophagy inhibition with chemo‐dynamic therapy, thereby promoting immunogenic cell death [69]. This nanocomplex demonstrated enhanced antitumor efficacy when used in conjunction with aPD‐L1 immunotherapy.

More research focuses on clinical‐approved inhibitors such as HCQ and CQ for potential clinical applications. In clinical settings, HCQ and CQ require high doses and have serious side effects, limiting their clinical applications [70]. By employing bio‐nanotechnology, chemical autophagy inhibitors could be co‐loaded with other therapeutic agents, achieving combined therapies [71]. It is worth mentioning that Ji, et al. designed an optimal co‐delivery nanoformulation for targeted drug palbociclib (PAL) and HCQ in pancreatic ductal adenocarcinoma treatment [72]. The use of HCQ in combination with CDK4/6i PAL has demonstrated a decreased stress‐tolerance response. After this, they found the repetitive dose of co‐delivery lipid‐coated mesoporous silica NP activates the anti‐apoptotic pathway Bcl‐2, while adding another Bcl‐2 inhibitor (ABT‐737) further improved the performance of co‐delivery nanoparticles. In another study, Gu, et al. developed a pH‐responsive core‐shell nanocarrier, CP@NP‐cRGD, in combination with the targeted EGFR tyrosine kinase inhibitors (TKIs) AZD9291, for the treatment of NSCLC [73]. They co‐loaded CQ and PD173074 onto the NPs to reverse the resistance of AZD9291 through inhibiting autophagy and selectively inhibiting FGFR1, respectively. In‐vitro experiments demonstrated the unique sequential release characteristics of CP@NP‐cRGD, with CQ exhibiting an initial burst effect followed by a sustained release and PD173074 showing a relatively slow release over time. In vivo, CP@NP‐cRGD induced more cell apoptosis and less proliferation by suppressing protective autophagy in NSCLC. Further investigation, transmission electron microscopy revealed fewer autophagosomes in the control group, while the AZD9291 group exhibited a significant accumulation of autophagosomes. Upon combination with CQ and PD173074, further accumulation of autophagosomes and a decrease of autolysosomes were observed. This phenomenon may be attributed to reduced autophagosome fusion (autophagy inhibition).

Aside from this, gene therapy has been incorporated as well. The co‐delivery of various nucleic acid molecules (e.g., siRNA, miRNA, and shRNA) strongly increased the anti‐tumor efficacy of clinical drugs by inhibiting autophagy, which could be an alternative for tumor combined therapy [74]. A novel chimeric peptide supramolecular nanoparticle delivery system was developed by Wu, et al., enabling the specific delivery of miR‐9 along with a targeting peptide to pancreatic ductal adenocarcinoma (PDAC). In their study, miR‐9 inhibits autophagy by downregulating eIF5A2 expression, suppressing autophagy in PDAC treatment and inducing cell apoptosis. Significantly, the overexpression of miR‐9 enhances the sensitivity of PDAC cells to doxorubicin and serves as a potential biomarker for predicting doxorubicin chemoresistance [74a]. Combining drug therapy with the delivery of autophagy inhibitory genes represents an attractive novel therapeutic approach for cancer treatment. However, its widespread clinical application has not yet been achieved due to the lack of identified optimal therapeutic targets across different types of tumors.

#### 3.1.2 Sensitizing Tumor for Radiotherapy

Radiation therapy is one of the primary non‐surgical modalities in cancer treatment, with extensive applications in nanomedicine combined therapies [75]. More than half of cancers can be treated with radiotherapy, such as glioblastoma, prostate cancer and non‐small cell lung cancer [76]. The acquired resistance of cancer cells to radiotherapy, resulting in tumor recurrence and metastasis remains a significant clinical problem [77]. Notably, autophagy activation can overcome nutritional stress and resist radiation‐mediated cell damage [78]. Ma, et al. found that although nanomaterials based on gold nanospikes (GNS) can exhibit a significant dose‐dependent radiosensitization effect, autophagy induced by combining GNS and radiotherapy plays a protective role in radiotherapy [79]. Therefore, the appropriate combined use of autophagy inhibitors and nanomaterials may be an effective strategy to increase the sensitivity of cancer cells to ionizing radiation in cancer radiotherapy. Wu, et al. combined the autophagy inhibitor 3‐MA with silver nanoparticles AgNPs, and found that the essence of radiosensitization is to increase reactive oxygen species (ROS) [80]. AgNPs can induce a radiosensitizing effect by increasing ROS, while ROS‐activated autophagy inhibits the level of ROS. So, inhibiting autophagy with 3‐MA can further increase cellular ROS levels and improve radiotherapy's effectiveness.

**FIGURE 9:**
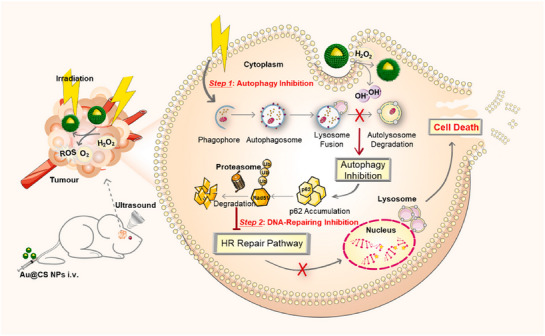
Schematic illustration of Au@CS NPs improving the radiotherapy efficacy by inhibiting the autophagy flux and hindering the homologous recombination repair pathway. Reproduced with permission [82]. Copyright 2022, Elsevier.

Inspired by the new strategy, autophagy inhibitors can be assembled in nanoparticles or directly use nanomaterials with autophagy inhibitory effects to achieve the “integrated” therapeutic effect of sensitization and autophagy inhibition. For example, the superparamagnetic nanoparticles (SPION) Fe_3_O_4_@AgNPs enhanced radiosensitivity by directly inhibiting early cytoprotective autophagy and increasing calcium‐dependent apoptosis in later stages [81]. A similar NP, core‐shell copper selenide‐coated gold nanoparticles (Au@Cu_2−_ *_x_* Se NPs), were designed to inhibit protective autophagy and DNA repair of tumor cells in radiotherapy [82]. Au@Cu_2−_ *_x_* Se NPs inhibited autophagy by altering the pH of lysosomes in tumor cells, thereby suppressing the formation of autolysosomes. Additionally, they can further enhance the critical DNA repair enzyme Rad51 in the homologous recombination repair pathway to prevent DNA damage repair in tumor cells (Figure 9).

**FIGURE 10:**
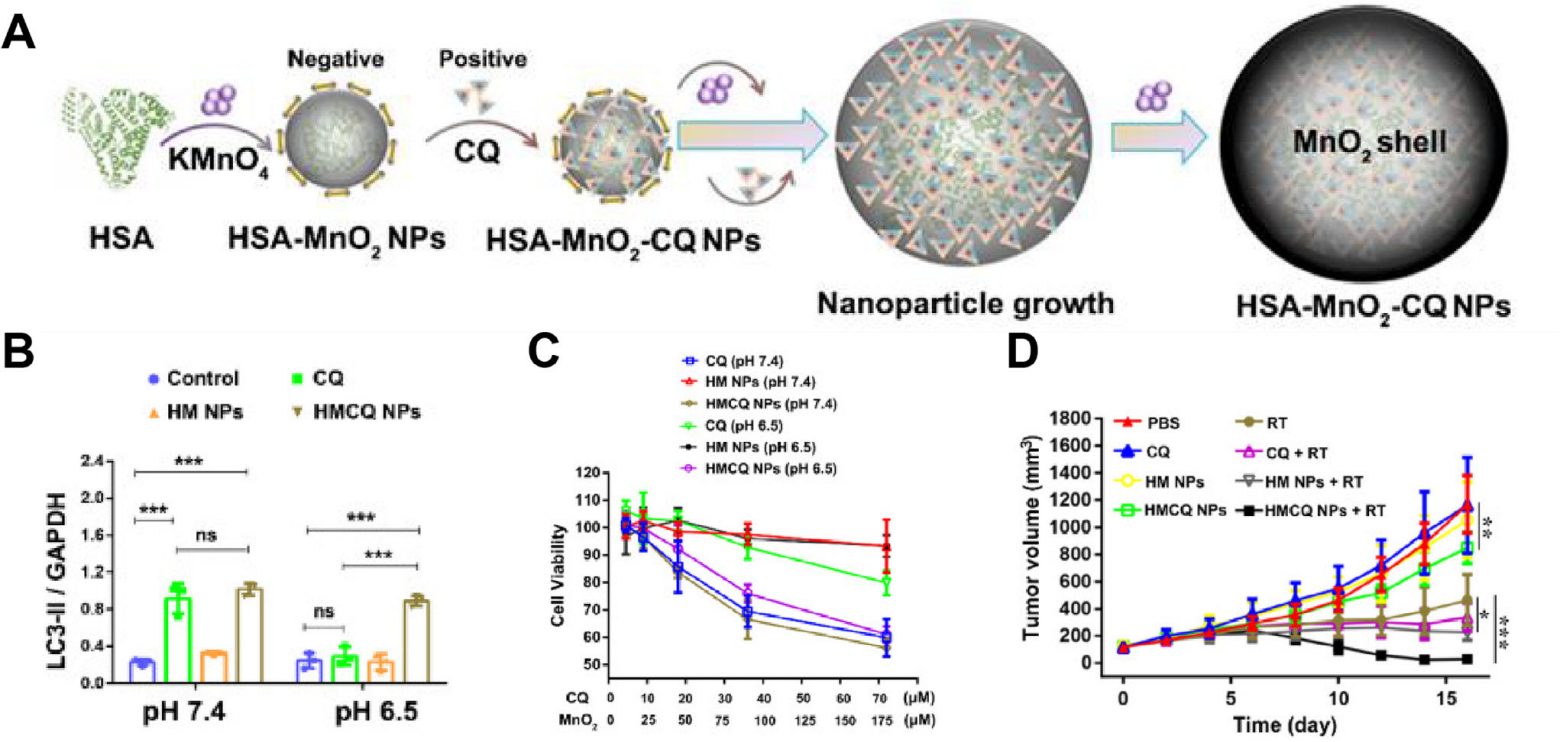
(A) Schematic diagram depicting the preparation of HSA‐MnO_2_‐CQ nanoparticles. (B, C) Acidic conditions inhibit the effect of CQ, and HSA‐MnO_2_‐CQ NPs restored CQ's autophagy inhibition ability and cytotoxicity. (D) Tumor growth curves after different treatments in the presence or absence of radiation. Reproduced under the terms of the CC BY 4.0 license [84]. Copyright 2020, Copyright Lin, et al.

In addition, loading autophagy inhibitors can exert an even stronger autophagy inhibition effect than naked nanomaterials. Li, et al. synthesized hollow mesoporous silica nanoparticles (HMSN) loaded with HCQ [83]. The advantage of HMSN is that it can be prepared on a large scale into uniformly distributed agents, and its drug release kinetics can be controlled by regulating its pore size. HMSN also enables HCQ to accumulate in tumors and prevent non‐specific side effects by enhancing permeability and retention (EPR) effects. They found that the ratio of LC3‐II to LC3‐I in HCQ‐HMSN‐treated cancer cells was higher than that in HCQ‐treated cells, especially after irradiation, suggesting a stronger autophagy inhibitory effect. When comparing the treatment effect of HCQ‐HMSN+RT combined group and RT treatment group, the average tumor size of the former was reduced by 70.8%. Based on drug delivery, Lin, et al. designed a more ingenious controlled drug release system, which utilized the strong charge force binding between positively charged CQ and negatively charged manganese dioxide (MnO_2_) nanoparticles and co‐deposited on the template of nanoparticles with human serum albumin (HSA) as carriers (Figure 10A) [84]. After exposure to the acidic tumor microenvironment, it reacts with H^+^/H_2_O_2_ to generate O_2_ and raise the pH of the TME, and then releases the encapsulated CQ in a H^+^/H_2_O_2_ concentration‐dependent manner. The generated O_2_ alleviates hypoxia‐induced autophagy, while elevated pH increases the cellular internalization efficiency of CQ, and inhibits autophagy more effectively in cells (Figure 10B,C). In vivo, free CQ and HSA MnO_2_ NP do not affect tumor growth. In contrast, HSA MnO_2_‐CQ NP achieves 27.5% inhibition of tumor growth, and 97.5% of inhibition in tumor growth was observed in the HSA‐MnO2‐CQ NPs + RT combination treatment (Figure 10D).

#### 3.1.3 Enhancing PTT/PDT

PTT/PDT involves the irradiation of specific light wavelengths, leading to collisions between photosensitizers and surrounding molecules. This process results in a mild, brief elevation in tissue temperature, capable of inducing various effects, such as promoting blood flow, which can lead to tumor reoxygenation (between 39 and 44°C), preventing DNA damage repair (>41°C), and activating anti‐tumor immune responses [85]. Photothermal therapy uses energy to enhance the heating of cells and tissues in local areas, causing irreversible damage and cell death [86]. However, there are limitations to this therapy. On the one hand, high temperatures above 50°C are usually required to ablate tumors and nearby normal tissue, which could decrease the risk of recurrence or metastasis [87]. On the other hand, heat stress induces protective autophagy and limits the efficacy of PTT [88].

In response to these problems, researchers first considered loading autophagy inhibitors such as CQ and primaquine (PQ) on nanoparticles [89]. For example, Chen, et al. embedded CQ‐loaded bismuth crystals into hollow SiO_2_ nanoparticles. Applied SiO_2_ prevented the oxidation of bismuth crystals and achieved a high photothermal conversion rate. Meanwhile, CQ molecular significantly weakened the degradation of autolysosomes by lysosome within the tumor cells, thus inducing a suppression effect to autophagy and resistance to photothermia [89b].

**FIGURE 11:**
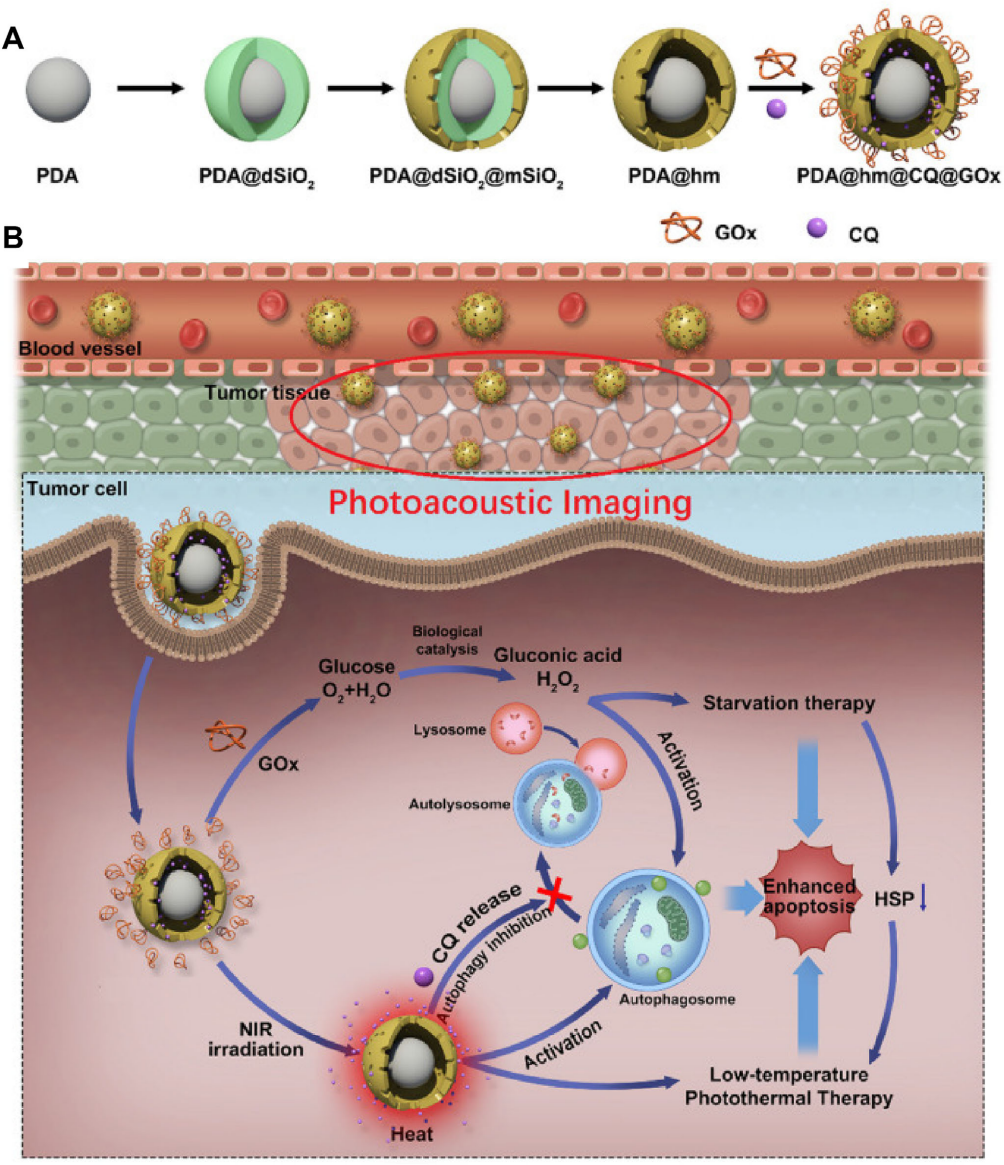
(A) Schematic illustration of corona‐like structure PDA@hm@CQ@GOx nanoparticles. (B) PDA@hm@CQ@Gox can inhibit the autophagy and expression of HSP induced by photothermal therapy. Reproduced under the terms of the CC BY 4.0 license [93]. Copyright 2020, Copyright Shao, et al.

A new strategy has been developed to effectively destroy tumors under mild conditions (below 45°C) [87, 90]. Nevertheless, the autophagy process will also be activated under mild‐temperature PTT treatment via the effects of heat shock proteins (HSPs) [91]. The main solution is combining photothermal agents with HSP or autophagy inhibitors [92]. Shao, et al. loaded chloroquine (CQ) and the glucose consumer glucose oxidase (GOx) into separate polydopamine compartments and decorated the surface of hollow silica nanoparticles to form a multi‐functional tambourine‐like nanostructure (Figure 11A) [93]. Initially, surface‐bound GOx mediated tumor starvation, directly suppressing the expression of HSP70 and HSP90, thereby enhancing the low‐temperature photothermal therapy (PTT) induced by the PDA nano‐core. Furthermore, the subsequent release of CQ inhibited autophagy to compensate for the compensatory autophagy activated by PTT and starvation (Figure 11B). Using the same principle, as mentioned earlier, Cui, et al. incorporated the HSP70 small‐molecule inhibitor apoptozole with the photosensitizer ICG on protamine (PS)‐modified NDs. NDs can act as an effective autophagy inhibitor, enhancing mild‐temperature photothermal therapy with apoptozole [67].

Building upon these challenges, some researchers have harnessed PDT as a potent strategy for enhancing immunotherapy against refractory cancers, a concept referred to as photoimmunotherapy [85]. In this context, the team led by Xiao employed a lipid‐based nanomaterial to encapsulate hydrochloride doxycycline and the photosensitizer chlorin e6 separately within the hydrophilic and hydrophobic layers [94]. Utilizing Doxy as an autophagy inhibitor, they not only mitigated treatment resistance but also upregulated MHC‐I expression, resulting in more effective antigen presentation and cytotoxic T lymphatics recognition, thereby enhancing tumor immunogenicity. Additionally, this approach promoted DC maturation and T‐cell maturation, reshaping the tumor immune‐suppressive microenvironment.

#### 3.1.4 Improving SDT

The sonodynamic therapy (SDT) principle is to apply ultrasonic cavitation to trigger specific sonochemical reactions to produce local cytotoxicity by integrating sonosensitizer and low‐intensity ultrasound [95]. Although SDT demonstrates stronger tissue penetration ability and less damage to surrounding tissue [96], the application is limited by the selection of sonosensitizers, including organic and inorganic sonosensitizers. Moreover, many sensitizers, such as protoporphyrin, often have defects, including poor tumor enrichment, solubility and stability. Inorganic acoustic sensitizers such as titanium dioxide (TiO_2_) have high stability of light and more stable physicochemical characteristics. However, the insufficient sonosensitivity conversion rate and the long‐term toxicity still need to be solved [97]. In addition, autophagy induced by SDT is generally inadequate, but instead becomes a survival pathway for cancerous cells, leading to resistance to SDT treatment.

Given the above problems, the current research is considering combining sonosensitizers and autophagy inhibition with nanomaterials to achieve the goal of all‐in‐one sonodynamic therapy. Qu, et al. capitalized on the unique advantages of SDT in facilitating drug penetration across the blood‐brain barrier (BBB) and designed an all‐in‐one nanosensitizer [98]. They integrated the sonosensitizer chlorin e6 (Ce6) and the autophagy inhibitor HCQ into liposomes modified with the vascular iopep‐2 peptide. Upon ultrasound‐targeted microbubble disruption of the BBB, the NPs selectively accumulated in glioblastoma. Subsequently, the nanosensitizer responded to a second ultrasound stimulation, concurrently releasing HCQ and generating ROS in glioblastoma cells to inhibit mitochondrial autophagy. The inhibition of autophagy enhanced SDT‐induced cancer cell apoptosis. Feng, et al. developed a TiO_2_‐based biomimetic nanoplatforms [99], by loading HCQ inside and covering the surface with a cancer cell membrane coating, affording the NPs immune escape and tumor‐targeting ability. So nanoparticles could release HCQ and block autophagy flux in response to ultrasonic stimulation at the malignant region. The tumor growth curves and mice photos showed that either SDT or HCQ displayed a moderate inhibition of cancerous cell growth with the *v*/*v*_0_ of 3.68 ± 0.14 and 4.87 ± 0.22, respectively. In comparison, a marked decrease in the tumor volume with the *v*/*v*_0_ of 1.71 ± 0.11 was found in the combined group, exhibiting an excellent therapeutic effect. Zhou, et al. co‐encapsulated the autophagy inhibitor 3‐MA and the sonosensitizer protoporphyrin IX (PpIX) in liposomes to achieve all‐in‐one sonodynamic therapy [100]. High‐throughput RNA sequencing was conducted to investigate the impact of SDT on autophagy, and the results revealed that differentially expressed mRNA was associated with autophagy‐related MAPK and AMPK signaling pathways.

### 3.2 Autophagy‐Inducing Nanomedicine Applied in Combined Therapies

**FIGURE 12:**
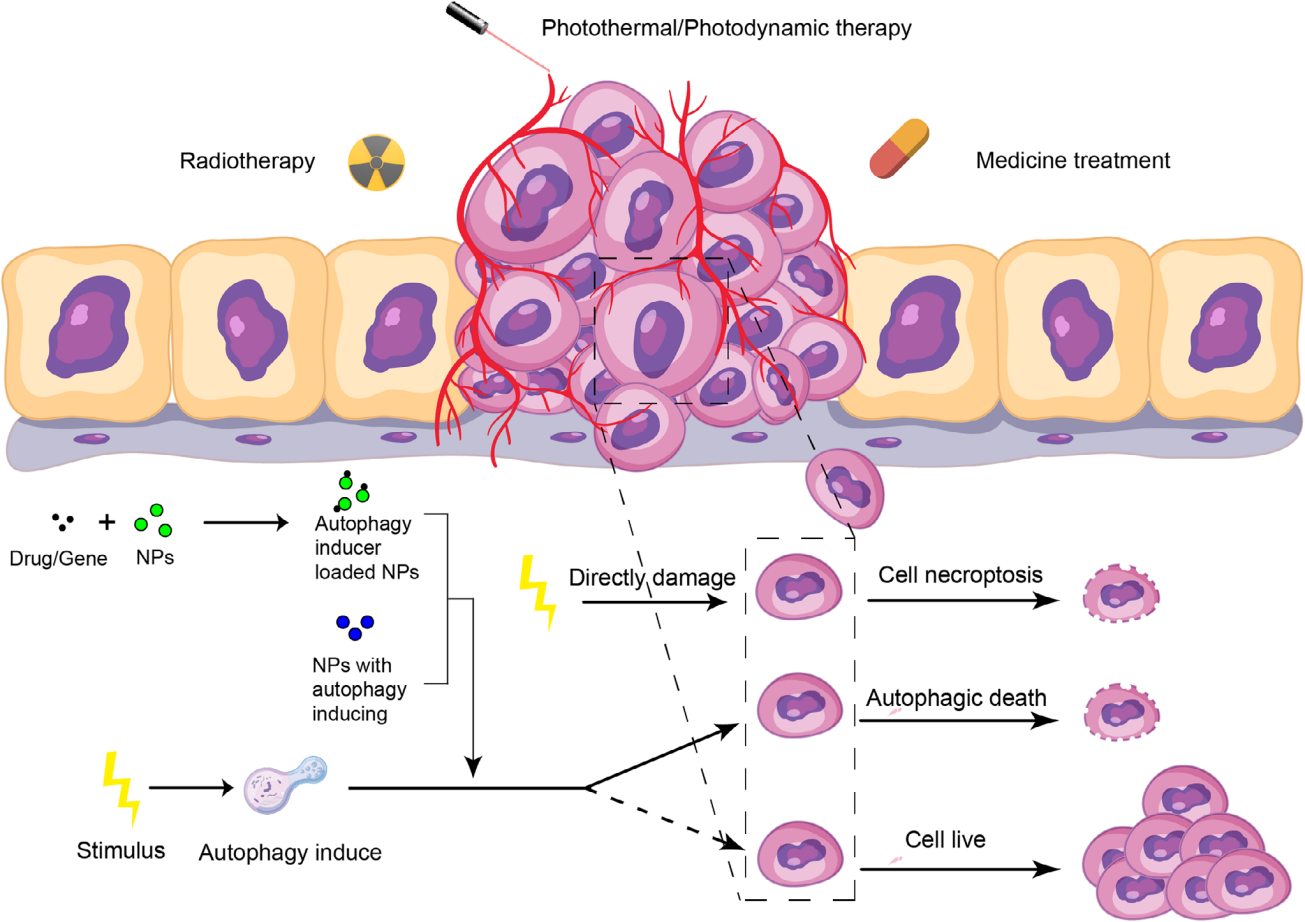
Schematic of combined treatment of anti‐tumor with nanoparticles inducing autophagy.

The dynamic cellular process of autophagy in cancer cells serves dual functions, promoting both cell survival and cell death. In the pursuit of innovative cancer treatment approaches, incorporating autophagy‐inducing nanomedicine into combined therapies presents a promising opportunity to improve treatment effectiveness (Figure 12). A comprehensive summary of nanomedicine strategies that leverage autophagy induction to enhance chemotherapy, radiotherapy, phototherapy, or immunotherapy is presented in Table 4.

**TABLE 4:** Representative autophagy‐inducing nanomedicine applied in combined therapies.

Type of NPs	NPs	Size	Drug/gene delivery	In vitro/in vivo	Cell/animal model	Combination therapy	Major outcomes	Ref.
Carbon‐based nanoparticles	FePt/GO nanosheets	500 nm	—	In vitro, in vivo	HeLa, H460, HELF, A549, H1975 and LLC cells, LLC tumor bearing mice	Radiotherapy	FePt/GO NSs selectively inhibit cancer cell proliferation and induce autophagy, which may contribute to radiotherapy sensitivity	[101]
Inorganic nanoparticles	Hydroxyapatite nanoparticles (B‐SeHANs)	500 nm	Selenium	In vitro, in vivo	MNNG, HOS and bMSCs cells, osteosarcoma orthotopic xenograft mouse model	Chemotherapy	The Bone‐mimetic hierarchical construction significantly enhanced SeHANs‐induced apoptosis and autophagy, thereby inhibiting tumor growth and reducing tumor‐induced bone destruction.	[123]
Lipid‐based nanoparticles	Nanoliposomes	106.35 ± 2.25 nm	IR780, chlorophyll‐rich fraction of Anthocephalus cadamba (CfAc)	In vitro, in vivo	MCF‐7, MCF‐10A, 4T1, L929 and NIH3T3 cells, 4T1 orthotopic mouse model	Photothermal therapy	Bioactive nanoliposomes containing NIR dyes and phytopharmaceuticals enhance ROS production under NIR light, leading to autophagic death of cancer cells upon NIR stimulation	[105]
Metal nanoparticles	Cu‐Cy nanoparticles	—	—	In vitro	SW620 cells	Radiotherapy	X‐ray irradiation of Cu‐Cy nanoparticles to induce autophagy may be the mechanism by which they act as radiosensitizers	[102]
	GNs	40 nm	Anti‐EGFR antibody	In vitro, in vivo	MDA‐MB‐231, Xenograft Tumor Model	Photothermal therapy	Anti‐EGFR‐GNs combined with NIR‐PTT promotes autophagy by increasing beclin‐1, ATG5, p62 and LC3 and inhibiting AKT/mTOR signaling pathway, which may serve as an alternative cell death mechanism	[106]
	Polydopamine‐coated branched Au‐Ag nanoparticles	200 nm	—	In vitro, in vivo	T24 and Hela cells, Xenograft nude tumor model	Photothermal therapy	The NPs could inhibit the proliferation of T24 cells by altering the expression of cyclin A and p21 to cause S phase arrest, leading to cell death by inducing mitochondria‐mediated cellular apoptosis and increasing LC3‐II protein expression and LC3 punctate foci to trigger autophagy.	[107]
	Ag@ZnO nanoparticles	70 nm	—	In vitro	A375, normal dermal fibroblasts cells	Photodynamic therapy	Increased intracellular ROS production triggered by Ag@ZnO NPs under UV irradiation induces stress‐related Golgi fragmentation and autophagy, ultimately leading to melanoma cell apoptosis	[116]
	Iron‐palladium (FePd) nanocrystal	105 nm		In vitro, in vivo	4T1 and 293T cells, 4T1 subcutaneous tumor model, 4T1 lung metastasis tumor model.	Immunotherapy	Engineered FePd nanocrystals with spiky morphology enhanced autophagy and ferroptosis in cancer cells. These nanocrystals also promoted immune activation, improving the efficacy of anti‐PD‐L1 immunotherapy.	[121]
Metal oxide nanoparticles	N‐TiO2 NPs	20–120 nm	—	In vitro	A375, H‐Fib	Photodynamic therapy	Low doses of N‐TiO_2_ NPs (1–100 µg mL^−1^) stimulate a autophagy flux response to promote survival. Only by blocking autophagy flux by producing a large amount of ROS under photo‐activation can necroptosis be induced	[114]
	ZnO NPs	172 nm	—	In vitro, in vivo	HeLa, 4T1 and MCF‐7/ADR cells, 4T1 orthotopic mouse model	Chemotherapy	ZnO NPs‐induced autophagy enhances zinc ion release and ROS generation, promotes cancer cell death, and enhances tumor chemotherapy	[120a]
	Magneto‐gold@fluorescent polymer nanoparticle	30 nm	—	In vitro, in vivo	HepG2, HUVEC, xenografted tumor model	Chemotherapy	This nanoparticle can be efficiently absorbed and used simultaneously for in vivo tumor‐targeted T1 and T2‐MRI/CT/NIR fluorescence bioimaging and enhanced autophagic flux to enhance non‐toxic concentrations of doxorubicin Cancer Treatment with DOX	[120b]
Polymers	Polydopamine nanoparticles	101.96 ± 6.70 nm	Beclin 1‐derived peptide	In vitro, in vivo	MDA‐MB‐231, NIH3T3 and Hela	Photothermal therapy	Beclin 1‐derived peptide upregulates autophagy in cancer cells and further sensitizes tumors to photothermal ablation, enhancing the efficiency of photothermal therapy	[104]
	Dendrimer nanoparticles	66 nm	Rapamycin, phthalocyanine	In vitro, in vivo	A549 and 4T1 cells, Xenograft nude tumor model	Photodynamic therapy	Rapa, an autophagy inducer, significantly enhanced the activity of PDT against cancer cells, and the combination therapy of co‐loading Rapa and Pc in ROS‐sensitive self‐assembled dendrimer nanoparticles greatly enhanced the tumor suppression efficiency	[113]
	C‐TFG micelle	150 nm	STF‐62247, oxaliplatin prodrug	In vitro, in vivo	CT26 cells, CT26 tumor‐bearing mice	Chemoimmunotherapy	C‐TFG micelle can sensitively respond to oxaliplatin‐induced autophagy and release a powerful autophagy inducer STF‐62247, which precisely transforms autophagy to “overactivated” condition, leading tumor cells to autophagic death	[117]
	Polymeric nanoparticles (PEI‐GA)	102 ± 19 nm	DOX, shAkt1	In vitro, in vivo	HepG2 and Hepa‐1.6 cells, Xenograft Model Mice	Chemotherapy	PEI‐GA/DOX/shAkt1 has good tumor targeting ability, induces excessive autophagy through PI3K/Akt/mTOR signaling pathway to induce type II cell death, and increases the sensitivity of tumor cells to chemotherapy	[119a]
	Polycaprolactone microparticles	—	5‐fluorouracil	In vitro	CAL27, HSC3, and normal fibroblasts	Chemotherapy	MPs increase autophagy and DNA damage through PARP1 hyperactivity and lipidation of LC3‐II, which in turn increases cell death	[119b]
	Polymeric nanoparticles (CS/PAA/VP‐16@TPGS/PLGA NPs)	300 nm	Etoposide (VP‐16)	In vitro	A549 cells	Chemotherapy	CS/PAA/VP‐16@TPGS/PLGA NPs releases chemotherapeutic drugs in response to tumor pH and enhances the anticancer effects of drugs by inducing apoptosis and autophagy in MDR cells	[119c]
	Polymeric nanoparticles (PEI‐PLGA‐MNPs)	80 nm	Paclitaxel	In vitro	U251 cells	Chemotherapy	FITC‐labeled PEI‐PLGA‐PTX‐MNPs can be efficiently endocytosed by targeted U251 cells for cell imaging, inhibition of cell growth and migration, induction of apoptosis and autophagy.	[119d]
	Nanocomposite gel	38.3 ± 1.4 nm	Paclitaxel and temozolomide	In vitro, in vivo	C6 cells, orthotopic glioma model	Chemotherapy	Heat‐responsive nanocomposite gels rapidly gelled in vivo, sustained and slowly released PTX and TMZ, and induced autophagic cell death to exert antitumor effects	[119e]
	Polymeric nanoparticles (STF@AHMPP, EPI@AHMPTP)	225.7 ±0.5 nm, 231.4 ± 3.3 nm	STF‐62247, Epirubicin	In vitro, in vivo	L929 and CT26 cells, CT26 tumor‐bearing mice	Chemoimmunotherapy	The nanoplatform, utilizing an arginine fragment and hyaluronic acid skeleton, actively targeted tumor sites and released Epirubicin and the autophagy inducer STF‐62247, in combination with a PD‐L1 inhibitor. By inducing autophagic cell death, it enhanced the efficacy of both chemotherapy and immunotherapy in tumor treatment.	[122]
Semiconductor nanoparticles	Cu_(2−_ *_x_*_)_S nanocrystals	5–8 nm	—	In vitro	HepG2, HeLa, HUVECs and 3T3 cells	Photothermal therapy	d‐Cu_2−_ *_x_* S NCs can induce more vigorous cellular uptake and subsequent autophagy, resulting in higher cell ablation under near‐infrared light	[108]

Abbreviation: NIR: near infrared; ROS: reactive oxygen species; Cu‐Cy: copper cysteamine; GNs: gold nanorods; PTT: photothermal therapy; PDT: photodynamic therapy; N‐TiO_2_ NPs: nitrogen‐doped titanium dioxide nanoparticles; DOX: doxorubicin; PTX: paclitaxel; TMZ: temozolomide.

#### 3.2.1 Promotion of Radiotherapy Therapeutic Outcome

**FIGURE 13:**
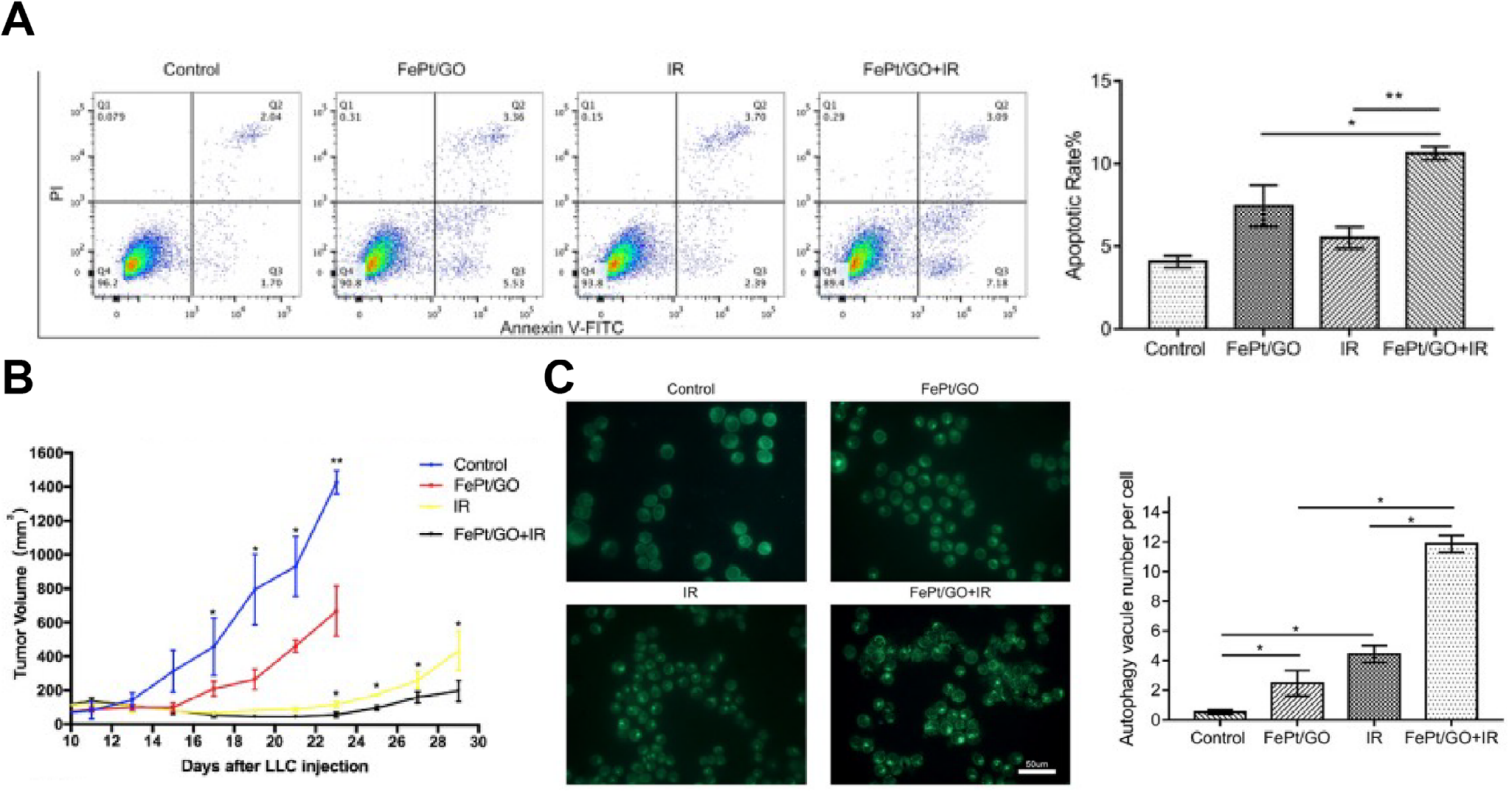
(A, B) FePt/GO increased cancer cells radiosensitization in vitro and vivo. (C) The number of autophagic vesicles in tumor cells increased when exposed to FePt/GO NSs combined with X‐ray beam. Reproduced under the terms of the CC BY‐NC 4.0 license [101]. Copyright 2019, Copyright lvyspring International Publisher.

Inhibition of autophagy could promote radiotherapy's therapeutic effect more than the strategy via autophagy induction, with only a few studies. Ma, et al. found that FePt/GO nanosheets selectively suppressed cancer cell proliferation and increased their radiosensitization in vitro and vivo (Figure 13A,B) [101]. Ma, et al. also observed autophagosomes increased when FePt/GO nanosheets were under an X‐ray beam (Figure 13C). The synergic effect was consistent with increased cytotoxicity, which implied a relationship between autophagy and synergic development. Thus, autophagy may be an essential link for FePt/GO NSs increased radiation sensitivity. As a new novel radiosensitizer, copper cysteamine (Cu‐Cy) nanoparticles can also efficiently improve X‐ray radiation to destroy colorectal cancer cells via the autophagy induced by X‐ray‐activated Cu‐Cy nanoparticles [102]. Liu, et al. utilized electron microscopy to observe morphological changes in cells and found that significant autophagosome formation was observed in SW620 cells after 1‐h co‐treatment with X‐rays and Cu‐Cy nanoparticles. Moreover, under X‐ray irradiation, Cu‐Cy nanoparticles exhibited a dose‐dependent cytotoxicity.

#### 3.2.2 Improvement of PTT/PDT by Upregulating Autophagy in Tumor Cells

**FIGURE 14:**
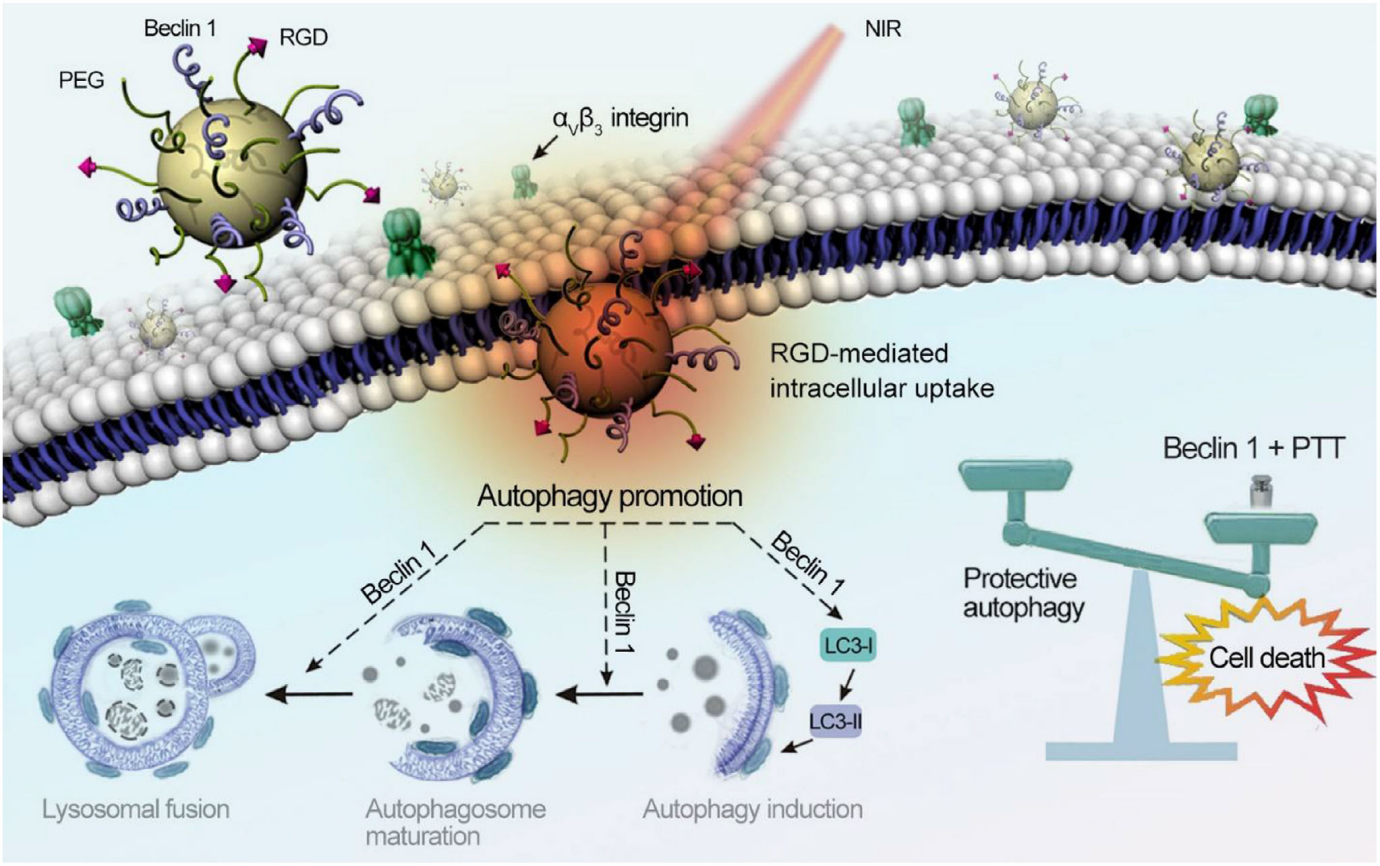
The illustration shows how beclin 1‐induced autophagy sensitizes cancer cells to photothermal killing. Cancer cells internalize PPBR through RGD‐αvβ3 recognition and the presence of beclin‐1 on the nanoparticle surface up‐regulates autophagy, making cancer cells more responsive to PTT. Reproduced with permission [104]. Copyright 2019, Elsevier.

In photothermal therapy, autophagy might play different roles in different cell types and with different photosensitizers [103]. In addition to blocking autophagy, induced autophagy can also be used to improve the therapeutic effect of PTT. Zhou, et al. fabricated a dual peptide decorated melanin‐like nanoparticle PPBR [104]. Beclin 1‐derived peptide modified on the polydopamine nanoparticle up‐regulated autophagy in cancer cells, and cyclic Arg‐Gly‐Asp (RGD) peptides decorated on the particle surface enhanced polydopamine nanoparticles' selectivity and cellular uptake. They observed autophagy induced by beclin‐1 might improve the therapeutic efficacy of PTT by destroying the autophagy's homeostatic functions and activating the autophagic cell death pathway (Figure 14). Appidi, et al. chose to encapsulate natural phytochemicals (a bioactive chlorophyll‐rich fraction of Anthocephalus cadamba (CfAc)) and light‐sensitive dye (IR780) in nanoliposomes form CIR NLP [105]. The CIR NLPs have a particle size of 106.35 ± 2.25 nm and a zeta potential of 11.62 ± 0.13 mV. Compared to free IR780, incorporating IR780 into CIR NLPs can enhance the absorbance intensity/stability. These bioactive nanoliposomes improve ROS generation, resulting in the autophagic death of cancer cells upon NIR stimuli without affecting the healthy cells/tissues.

Several metallic nanosystems have been studied for triggering autophagy during photothermal therapy. Zhang, et al. successfully synthesized anti‐EGFR antibody‐conjugated gold nanorods (anti‐EGFR‐GNs) and remarkably induced autophagy via combined NIR‐PTT, which resulted in EGFR‐targeted cancer cell death [106]. They also found that the cytotoxicity induced by anti‐EGFR‐GNs‐combined NIR‐PTT was rescued by treatment with 3‐MA, confirming the anti‐tumor effect of increased autophagy. In another study, however, polydopamine‐coated branched Au‐Ag nanoparticles (Au–Ag@PDA NPs) were found to inhibit the proliferation of human bladder cancer cells and induce cell cycle S arrest, apoptosis, and autophagy [107]. There is no direct evidence to prove the Au–Ag@PDA NPs caused autophagy, inhibiting tumor proliferation rather than protection.

A new point of view has been presented in a recent study. Chiral NPs may exhibit different cellular uptake, likely due to their different affinity with cytomembranes. D‐Cu_2−_ *_x_* S nanocrystals designed by Wang, et al. show more prominent cellular autophagy proceeds than L‐Cu_2−_ *_x_* S NCs [108]. Then, the cell ablation can be further enhanced by photothermal effects.

**FIGURE 15:**
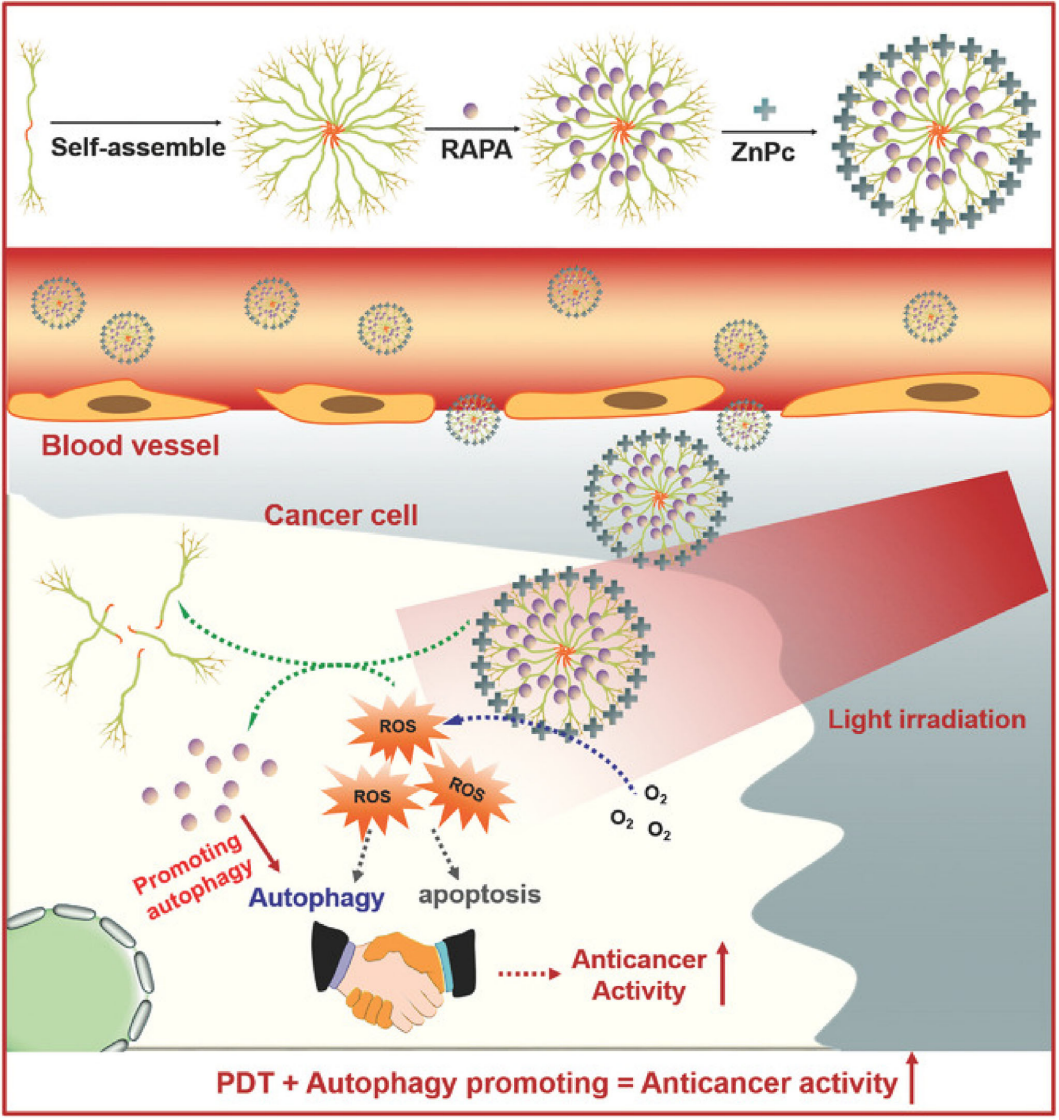
The schematic of the synthesis of ROS‐responsive dendrimer‐assembled nano‐carriers and the mechanism of promoting autophagy to enhance PDT treatment. Reproduced with permission [113]. Copyright 2018, John Wiley and Sons.

The application of PDT in skin diseases such as acne and keratosis has been extensively studied clinically [109], and in addition, several solid tumor types, including oesophageal, lung and prostate cancers, are viable targets for PDT [110]. Unlike PTT, PDT is predicated on generating reactive oxygen species (ROS) to induce cytotoxic effects [111]. It means that much stronger autophagy can be induced. However, which autophagy induced by PDT appears to play a prosurvival or a prodeath role remains a question [112]. To solve this problem, Wang, et al. used self‐assembled ROS response dendrimer vehicles (RSV) loaded with autophagy inhibitors (CQ, 3‐MA) and autophagy promoted (Rapa), respectively, and adsorbed the photosensitizer (phthalocyanine, Pc) on the RSV surface using electrostatic attraction [113]. After irradiation, the PDT process is triggered and ROS are released. Meanwhile, the thioacetal group of dendrimer nanoparticles was destroyed to release autophagy drugs (Figure 15). Finally, Wang group found that a synergistic combination of Pc and autophagy inducer Rapa greatly improved the tumor suppression efficiency, which proved that PDT‐induced autophagy was more of an anti‐tumor effect. Mohammadalipour, et al. also demonstrated that N‐TiO_2_ NPs (1‐100 µg mL^−1^) stimulate an autophagy flux response to promote survival at a low dosage [114]. Necroptosis can only be induced when autophagy flux was blocked via a large amount of ROS production under photo‐activation.

Metal Complexes can often be used instead of conventional photosensitizers in PDT processes. In combination of the autophagy‐inducing effect, metal nanoparticles are often used for PDT treatment of tumors [115]. For instance, core/shell NPs of Ag and ZnO (Ag@ZnO NPs) can be employed for UV‐inducible intracellular ROS generation, leading to autophagy and apoptotic cell death in melanoma cells [116].

#### 3.2.3 Synergistic Anti‐Cancer Effect via Drug Delivery

**FIGURE 16:**
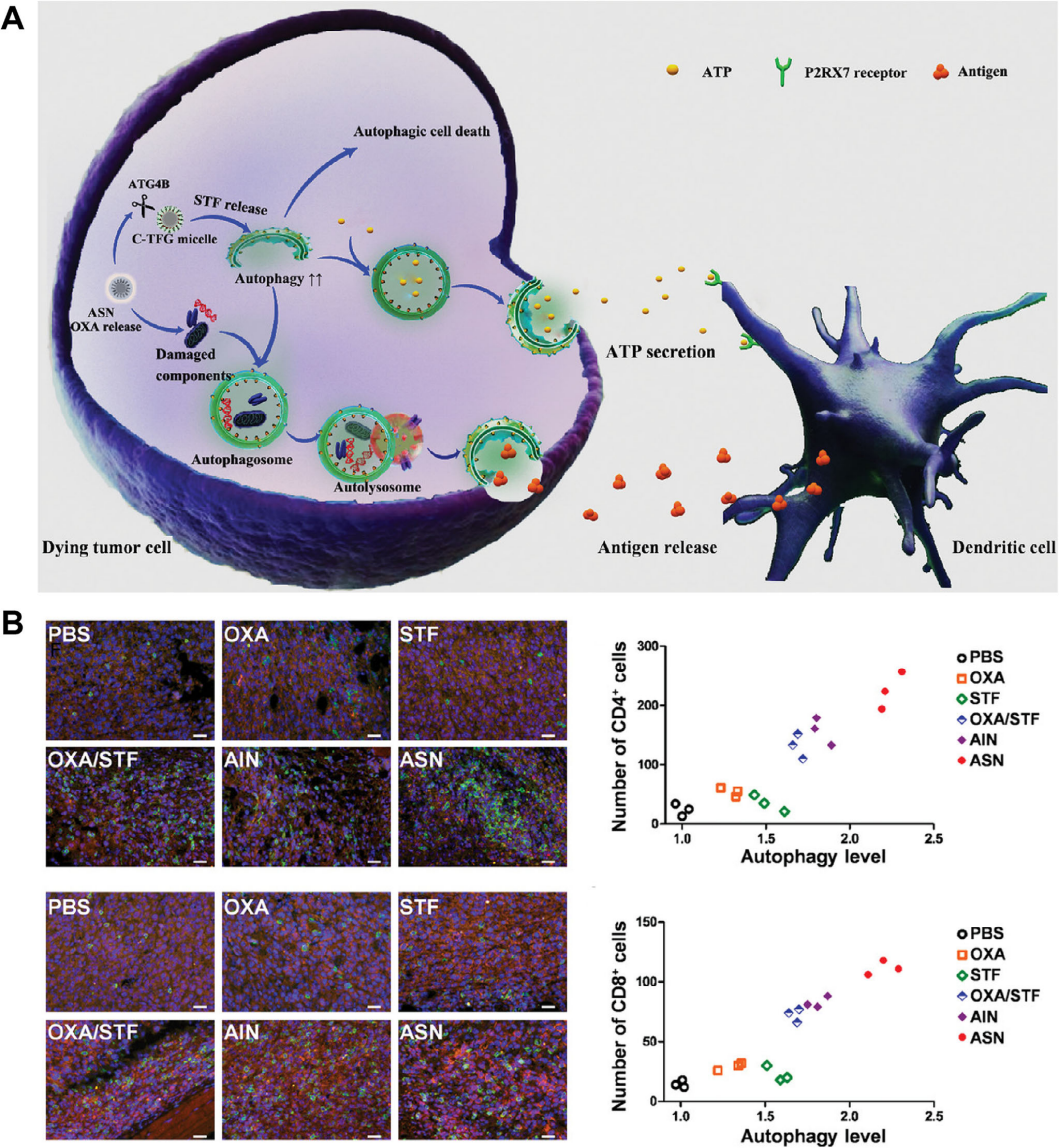
(A) Schematic of mechanism of OXA‐induced autophagic cell death and autophagy‐mediated DCs recruitment. (B) the immunofluorescence results and corresponding semi‐quantitative data showed that the tumor slices from the ASN group exhibited the highest level of autophagy and the greatest infiltration of immune cells, and the number of immune cells was positively correlated with LC3 level. Reproduced with permission [117]. Copyright 2020, John Wiley and Sons.

Different from the anti‐tumor mechanism of inhibiting protective autophagy from reversing the resistance to drug therapy, inducing overactivation of autophagy can directly cause type II apoptosis and kill tumor cells with anticancer drugs. Wang, et al. designed a type of autophagy‐sensitive nanoparticles (ASN), prepared by self‐assemble of autophagy‐sensitive micelles (C‐TFG micelles) and followed by electrostatic binding of oxaliplatin grafted hyaluronic acid prodrug (HA‐OXA) [117]. Once reaching the malignant tissues, the conjugated OXA would be reduced to free OXA under the intracellular reduction microenvironment to induce “mild activation” of autophagy. Subsequently, C‐TFG micelles disintegrated immediately in response to OXA‐induced autophagy, enabling the “on‐demand” drug release of the potent autophagy inducer STF‐62247, which stimulates “over‐activated” autophagy, leading to autophagic death of tumor cells (Figure 16A). The “overactivated” autophagy can also enhance subsequent tumor antigen processing of the dying cells. Co‐staining of LC3 and CD4 or CD8 in tumor sections by immunofluorescence method showed that tumor slices from the ASN group exhibited the highest level of autophagy and the number of CD4^+^or CD8^+^cells was positively correlated with the level of LC3 (Figure 16B). Long, et al. employed polymers to deliver STF‐62247 and epirubicin to tumor sites [118]. They demonstrated that the combination of STF‐62247 with epirubicin resulted in significant autophagy activation and induced immunogenic cell death. Other functionalized polymers, such as CS, hydrogel, PLGA, etc., have also been combined with chemotherapy to induce enhanced autophagy and promote anti‐tumor effects [119].

**FIGURE 17:**
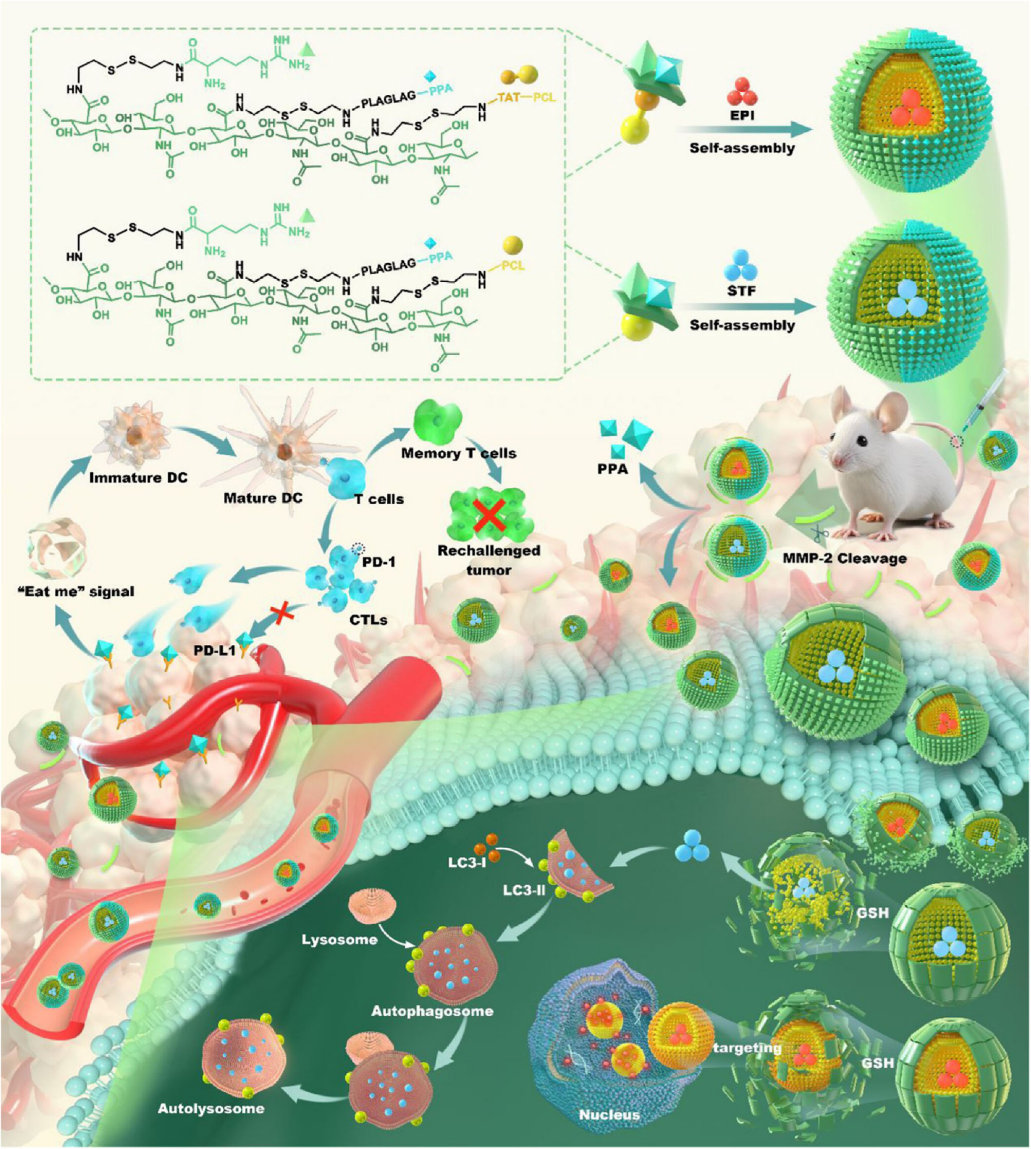
Redox‐triggered nanoplatform enables localized release of epirubicin and STF‐62247 at tumor sites, while PD‐L1 inhibitor D‐PPA enhances immune activation for combined chemotherapeutic immunotherapy. Reproduced with permission [122]. Copyright 2024, American Chemical Society.

Some studies take advantage of the autophagy‐inducing properties of metal nanoparticles, which are directly combined with chemotherapy or immunotherapy to suppress tumors [120]. Hu, et al. designed and synthesized a tetrapod spiky‐like iron‐palladium (FePd)‐polyvinylpyrrolidone (PVP) nanomedicine (TFPs) [121]. TFPs can mimic peroxidase and glutathione oxidase, inducing the accumulation of lipid peroxides at tumor sites and promoting ferroptosis in cancer cells. Moreover, the unique spiky topology effectively induce a strong autophagic response in cancer cells at low doses, significantly enhancing ferroptosis and triggering desirable immunogenic cell death. Importantly, this special topology effectively activates macrophages to release inflammatory cytokines, thereby augmenting aPD‐L1 immunotherapy. Li, et al. further developed a redox‐triggered autophagy‐inducing combination nanoplatform based on a hyaluronic acid scaffold [122]. This platform allows for the localized release of the chemotherapeutic agent epirubicin and the autophagy inducer STF‐62247 at tumor sites. Subsequently, the extracellular release of the PD‐L1 inhibitor D‐PPA peptide enhanced the activation of dendritic cells and cytotoxic T lymphocytes, thereby promoting tumor chemoimmunotherapy (Figure 17).

**FIGURE 18:**
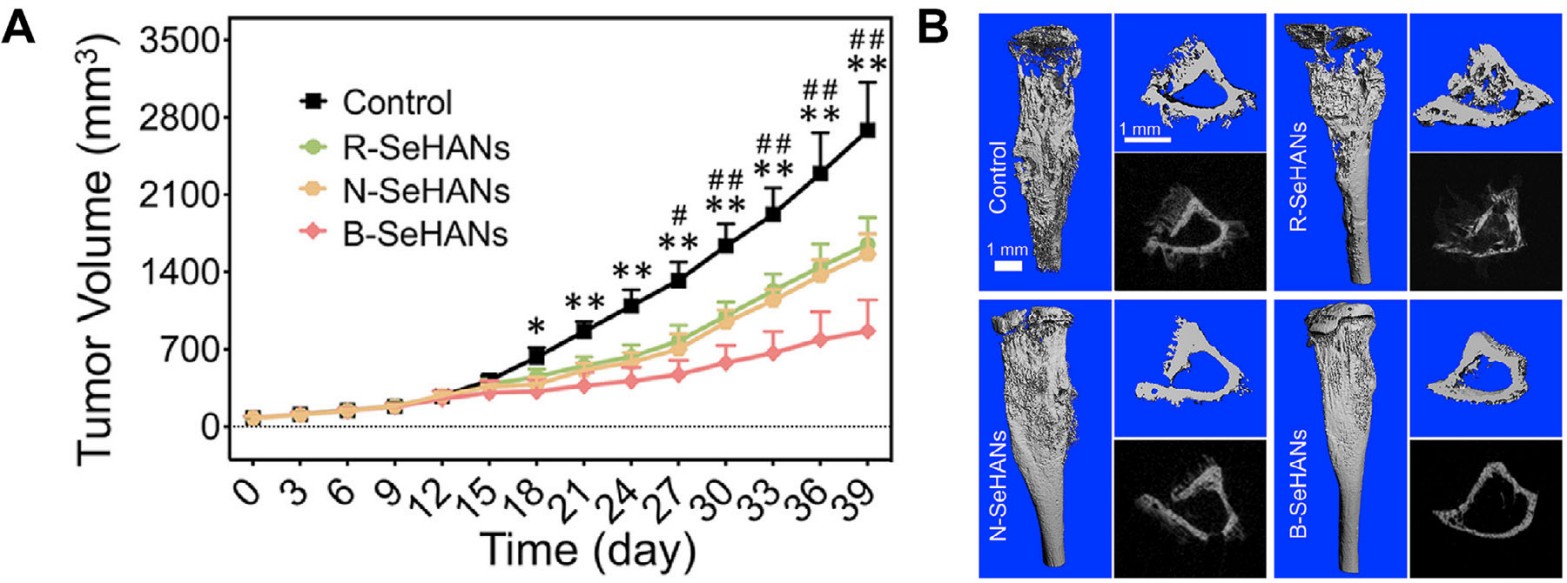
(A) In vivo evaluation of osteosarcoma growth inhibition. (B) Evaluation of bone destruction by microscopic CT images of the tibia of nude mice. Reproduced with permission [123]. Copyright 2020, Elsevier.

Li, et al. prepare biomimetic selenium‐doped hydroxyapatite nanoparticles (B‐SeHANs) by mimicking the uniaxially oriented hierarchical structure of bone hydroxyapatite [123]. The biomimetic NPs exhibited highly enhanced cellular internalization and induced autophagy through the JNK and Akt/mTOR pathways. Compared with the control group and rod‐like (R‐SeHANs) or needle‐like (N‐SeHANs) NPs, B‐SeHANs have more efficiently promoted both the formation of autophagosomes and the progression of autophagic flow. After 39 days of treatment, B‐SeHANs showed the most potent inhibition effect, inhibiting the tumor volume to about 865.86 mm^3^, which is much smaller than R‐SeHANs (1650.66 mm^3^), N‐SeHANs (1563.91 mm^3^) and control (2682.86 mm^3^) (Figure 18A). It is also found that B‐SeHANs can greatly protect the tibia from bone damage caused by tumors. Only a slight abrasive abnormal bone formation was observed on the tibia surface in B‐SeHANs group (Figure 18B).

## 4 Conclusion and Future Directions

Autophagy has a dual role in promoting and suppressing cancer, acting as a tumor suppressor in early stages and as a promoter for tumor maintenance and treatment resistance in advanced stages. In the early stage of tumorigenesis, autophagy serves as a survival pathway, providing biomaterials and energy to cope with stress, contributing to normal cellular physiological metabolism, and as a quality control mechanism to remove damaged proteins and organelles and prevent tumorigenesis. Conversely, autophagy can sustain metabolism and promote survival via nutrient recycling in the advanced stages of tumor development. Therefore, the regulation of autophagy can be used as an effective intervention strategy for preventing and treating cancer by preventing cancer development, limiting tumor progression and improving the efficiency of cancer treatment.

In contrast, autophagy often functions as a survival mechanism, and cancer cells can be favored by the cytoprotective effects of autophagy and become resistant to anticancer treatments. Therefore, inhibiting autophagy is a promising strategy to enhance cancer therapy when combined with other treatments.

Nanoparticles enable the targeted delivery of autophagy modulators at tumor sites, enhancing their potential to influence autophagy and regulate cancer progression. Nanoparticles are often engineered to release in response to specific stimuli for the targeted delivery of autophagy‐modulating drugs and related genes, which reduces off‐target effects of associated treatments. On the one hand, nanoparticles can aggregate specifically at the malignant region, have a sustained release effect on drugs, reduce the toxic and side effects, and maintain integrity and biological activity. On the other hand, nanoparticles enable efficient gene therapy by protecting genetic tools from degradation and increasing their uptake in tumor cells. In addition to drug and gene delivery in autophagy regulation, some nanoparticles can directly regulate autophagy. For example, nanodiamonds can directly act as autophagy inhibitors to regulate autophagy in cancer cells, which is beneficial to overcome therapeutic resistance in cancer treatment. However, it should be noted that the regulatory effect of some nanomaterials, such as TiO_2_‐NPs on autophagy is related to their physical properties.

We believe that the following key aspects in the field of autophagy‐targeted nanomedicine should be emphasized in the future, and we have summarized them in Table 5.

1. Which is more suitable for tumor treatment: inhibiting or inducing autophagy?

**TABLE 5:** Key points and recommendations.

Key points	Recommendations	Reasons
Direction of treatment	Inhibit autophagy	1. The mechanism of autophagic cell death remains unclear and its efficacy is variable 2. Inhibiting autophagy can overcome drug resistance
Type of tumor	Breast cancer, lung cancer, colorectal cancer, and pancreatic cancer	These tumor cells exhibit elevated levels of autophagy
Type of functionalization	Classic autophagy modulators	In close proximity to clinical application
	Novel autophagy regulatory molecules	More Innovative
Clinical application potential	Lipid‐based nanomaterials and polymer nanoparticles	1. Customizability; 2. Drug delivery ability; 3. Controlled Release; 4. Biocompatibility; 5. Obtained clinical approval and research verification

In this review, both strategies can effectively suppress tumor progression through distinct mechanisms. Inducing excessive autophagy can trigger autophagic cell death to kill cancerous cells. However, the outcomes of this approach in cancer treatment may vary depending on the cancer type, stage, and microenvironment. Despite extensive research, the mechanisms and regulation of autophagic cell death in cancer remain poorly understood, hindering its clinical translation.

In contrast, autophagy is predominantly regarded as a cellular survival mechanism. Cancer cells often develop resistance to conventional therapies such as chemotherapy and radiotherapy, and inhibiting autophagy can restore their sensitivity to various anticancer treatments. This implies that combining autophagy inhibitors with other anticancer agents can synergistically improve therapeutic efficacy. Therefore, therapeutic approaches targeting autophagy inhibition hold promising clinical prospects.

2. Which type of tumor therapy does autophagy‐targeted nanomedicine demonstrate the most promise?

This section primarily focuses on therapeutic approaches targeting autophagy inhibition, which have shown greater effectiveness in breast, lung, colorectal, and pancreatic cancer. This is attributed to elevated autophagy activity in these tumor types. For example, autophagy plays a pivotal role in supporting the survival and proliferation of breast cancer cells by providing nutrients and energy, particularly under stress conditions such as chemotherapy. This implies that inhibiting autophagy can disrupt this protective mechanism, rendering breast cancer cells more susceptible to the cytotoxic effects of chemotherapy drugs.

The heightened levels of autophagy observed in these tumors may be attributed to the activation of tumor‐specific receptors and specific gene mutations. For instance, ER‐positive breast cancer cells generally exhibit higher autophagy levels compared to ER‐negative cells. However, it is essential to note that autophagy levels can vary across different tumor subtypes and individuals, and the above observation represents a general trend.

3. What criteria and considerations should be employed to identify nanomaterials that offer distinct advantages and are well‐suited for the intended application?

In terms of inducing autophagy, unmodified silver and zinc oxide nanoparticles have effectively activated autophagy due to their small size and positively charged surface. These characteristics enable them to interact with the negatively charged cell membrane and facilitate better cell entry. Consequently, they can activate the autophagy pathway by inducing ROS‐mediated signaling. In terms of autophagy inhibition, nanodiamonds have demonstrated similar physical advantages. Nanodiamonds have been shown to inhibit autophagy by disrupting lysosomal function and affecting signaling pathways involved in autophagy regulation, such as the mammalian target of rapamycin (mTOR) pathway.

The utilization of naked nanoparticles alone is insufficient for achieving effective tumor‐specific autophagy modulation. Therefore, the functionalization of nanoparticles plays a critical role in enhancing their potential for targeted therapeutic interventions. (1) By surface modification, targeted delivery could be achieved and improve autophagy regulation in cancerous cells while minimizing any potential impact on normal cells. (2) Another approach involves the incorporation of molecules that regulate autophagy. Currently, most studies focus on incorporating classic autophagy modulators such as CQ, 3‐MA, and rapamycin into nanoparticles. These drugs have been widely used in clinical settings, making them more clinically relevant. This choice is driven by these drugs' clinical applicability, thereby enhancing the nanoparticle design's clinical suitability. (3) Furthermore, ongoing research aims to discover novel molecules for modulating autophagy. Researchers are exploring the incorporation of microRNAs, siRNAs, shRNAs, and autophagy‐regulating peptides that specifically target autophagy into nanomaterials. These investigations can potentially establish a new frontier in targeted autophagy nanomedicine.

4. Which specific types of nanoparticles show promising potential in future clinical applications for regulating autophagy?

The following nanoparticles may hold greater clinical translational significance. (1) Lipid‐based nanoparticles exhibit excellent biocompatibility and can encapsulate various drugs and nucleic acids. They have been used in formulating numerous FDA‐approved drugs. (2) Polymer nanoparticles offer the flexibility to be tailored with specific physicochemical properties to fulfill diverse therapeutic requirements. (3) Gold nanoparticles possess unique optical properties that make them suitable for imaging and photothermal therapy applications. (4) Iron oxide nanoparticles exhibit remarkable magnetic properties, rendering them suitable for contrast‐enhanced imaging and thermotherapy‐based cancer treatment.

Among them, lipid‐based nanomaterials and polymer nanoparticles are widely applied. Due to their high customizability, lipid‐based nanomaterials and polymer nanoparticles can encapsulate and stabilize various types of drugs, allowing for design and improvement tailored to specific application requirements. This enables controlled or targeted drug release, facilitating slow or site‐specific delivery. In addition, their remarkable biocompatibility has been clinically approved and validated through extensive research. Furthermore, substantial clinical studies have proven their effectiveness and safety. As a result, lipid‐based nanomaterials and polymer nanoparticles have emerged as the preferred nanocarrier systems for clinicians and researchers.

The next step is to consider translating preclinical knowledge of nanoparticle‐mediated autophagy regulation strategies for tumor therapy into clinical applications. However, there are still many problems for nanomaterials that should be solved. First, the large‐scale production and quality control of nanomaterials is challenging due to the difficulty of synthesis and high price. Second, the exact mechanism of autophagy in tumors is ambiguous, it is difficult to grasp the dual role of autophagy, and the side effects of autophagy regulation in nanotechnology platform‐based combination therapy are also unclear. Therefore, more mechanism studies, clinical trials, and safety and side effects evaluations must be carried out before clinical application.

## Author Contributions

Zhouyi Sun conceived and designed the framework of the review. Zhouyi Sun, Kai Zhang, Yang Liu, Qianwen Wang, Qitao Hu and Jiwei Qian conducted the literature search and organized the references. Zhouyi Sun and Huali Zuo wrote the initial draft of the manuscript. Bo Zhang, Zhe Tang, and Weiyu Chen supervised the manuscript. All authors contributed to the critical revision of the manuscript and approved the final version.

## Conflicts of Interest

The authors declare no conflicts of interest.
